# Comparative genomics of *Deinococcus radiodurans*: unveiling genetic discrepancies between ATCC 13939K and BAA-816 strains

**DOI:** 10.3389/fmicb.2024.1410024

**Published:** 2024-06-19

**Authors:** Soyoung Jeong, Harinder Singh, Jong-Hyun Jung, Kwang-Woo Jung, Sangryeol Ryu, Sangyong Lim

**Affiliations:** ^1^Radiation Biotechnology Division, Korea Atomic Energy Research Institute, Jeongeup, Republic of Korea; ^2^Department of Food and Animal Biotechnology, Seoul National University, Seoul, Republic of Korea; ^3^Department of Agricultural Biotechnology, Seoul National University, Seoul, Republic of Korea; ^4^Department of Biological Sciences, Sunandan Divatia School of Science, NMIMS Deemed to be University, Mumbai, India; ^5^Department of Radiation Science, University of Science and Technology, Daejeon, Republic of Korea

**Keywords:** *Deinococcus radiodurans*, comparative genomics, ATCC 13939, ATCC BAA-816, gene annotation, insertion sequence elements

## Abstract

The *Deinococcus* genus is renowned for its remarkable resilience against environmental stresses, including ionizing radiation, desiccation, and oxidative damage. This resilience is attributed to its sophisticated DNA repair mechanisms and robust defense systems, enabling it to recover from extensive damage and thrive under extreme conditions. Central to *Deinococcus* research, the *D. radiodurans* strains ATCC BAA-816 and ATCC 13939 facilitate extensive studies into this remarkably resilient genus. This study focused on delineating genetic discrepancies between these strains by sequencing our laboratory’s ATCC 13939 specimen (ATCC 13939K) and juxtaposing it with ATCC BAA-816. We uncovered 436 DNA sequence differences within ATCC 13939K, including 100 single nucleotide variations, 278 insertions, and 58 deletions, which could induce frameshifts altering protein-coding genes. Gene annotation revisions accounting for gene fusions and the reconciliation of gene lengths uncovered novel protein-coding genes and refined the functional categorizations of established ones. Additionally, the analysis pointed out genome structural variations due to insertion sequence (IS) elements, underscoring the *D. radiodurans* genome’s plasticity. Notably, ATCC 13939K exhibited a loss of six IS*Dra2* elements relative to BAA-816, restoring genes fragmented by IS*Dra2*, such as those encoding for α/β hydrolase and serine protease, and revealing new open reading frames, including genes imperative for acetoin decomposition. This comparative genomic study offers vital insights into the metabolic capabilities and resilience strategies of *D. radiodurans*.

## 1 Introduction

The order *Deinococcales*, part of the *Deinococcus*-*Thermus* phylum, consists of two families: *Deinococcaceae* and *Trueperaceae* (Ho et al., 2016). The *Deinococcaceae* family encompasses two genera: *Deinococcus*, which currently has 89 species with validly published names, and *Deinobacterium*, which only comprises one species, *Deinobacterium chartae* (Parte et al., 2020). *Deinococcus radiodurans* (*D. radiodurans*), the representative species of the *Deinococcus* genus, exhibits remarkable resilience to various stresses, including ionizing radiation (IR), UV exposure, DNA-damaging reagents, desiccation, and oxidative stress (Cox and Battista, 2005).

Since its discovery in 1956 as a contaminant in food sterilized by gamma radiation (γ-radiation) (Brooks and Murray, 1981), *D. radiodurans* (previously known as *Micrococcus radiodurans*) has been widely studied as a model organism to explore mechanisms underlying the extreme resistance to radiation and oxidative stress (Slade and Radman, 2011; Lim et al., 2019). Double-strand DNA breaks (DSBs), the most lethal form of DNA damage caused by IR, are repaired through the homologous recombination (HR) pathway. *D. radiodurans* utilizes synthesis-dependent strand annealing (SDSA), a primary HR subpathway for DSB repair (Zahradka et al., 2006; Elbakry and Löbrich, 2021). However, the presence of HR proteins with unconventional features, such as RecA and UvrD, combined with *Deinococcus*-specific proteins like DdrA and DdrB, amplifies the effectiveness of DSB repair (Bentchikou et al., 2010; Timmins and Moe, 2016). These combined actions potentially render the *D. radiodurans* version of SDSA, known as extended-SDSA (ESDSA), distinct from those of standard organisms. Besides traditional enzymatic antioxidants like catalase and superoxide dismutase, *D. radiodurans*’ radiation resistance is thought to be fortified by antioxidant complexes that combine manganese ions with metabolites, shielding proteins from oxidative damage (Daly et al., 2010). These findings suggest that the primary determinant of radiation resistance is proteome protection rather than genome preservation (Sharma et al., 2017). The distinct characteristics of this bacterium position it as an invaluable model for exploring various biological processes, including DNA repair, oxidative defense, and radiation biology.

*Deinococcus radiodurans* has also demonstrated potential applications in fields like bioremediation and biotechnology. This bacterium has been genetically tailored to remove heavy metals from radioactive locations or to possess enhanced biosorption capacity for uranium (Brim et al., 2000; Manobala et al., 2019). Biomaterials from *D. radiodurans*, including carotenoids (deinoxanthin), exopolysaccharides (DeinoPol), and membrane vesicles, exhibit significant industrial promise. Due to their antioxidant and radioprotective properties, these derivatives can suit food, cosmetics, and pharmaceutical industries (Farci et al., 2017; Jeong et al., 2020; Park et al., 2022; Han et al., 2023). The genetic materials of *D. radiodurans* have gained interest among researchers. By introducing the *D. radiodurans* regulators PprI (also called IrrE) and DR_1558 into industrial microbes, these microbes exhibit increased resistance to environmental stresses and produce more valuable natural compounds (Appukuttan et al., 2015; Park et al., 2020; Wang et al., 2024). A new RNA-guided nuclease suitable for genome editing was recently discovered in the *D. radiodurans* genome (Karvelis et al., 2021).

The *D. radiodurans* strain isolated in 1956 was named R1 and was designated ATCC 13939 at the ATCC. Its genome was sequenced in 1999 (White et al., 1999). However, it was discovered that the sequenced genome was not actually from the ATCC 13939 strain. Therefore, the sequenced R1 was re-designated as *D. radiodurans* R1 ATCC BAA-816 (White et al., 2004). Over the past two decades, several studies have noted genetic discrepancies between ATCC BAA-816 and ATCC 13939, resulting in a frameshift (Southworth and Perler, 2002; Mennecier et al., 2004; Cao and Julin, 2009; Niiranen et al., 2015). These genetic variations might have emerged due to separate laboratory cultivation (White et al., 2004). Since both strains have been instrumental in *Deinococcus* research, identifying the nucleotide sequence variations between ATCC 13939 and ATCC BAA-816 would be of significant interest to both the *Deinococcus* research community and those researchers studying DNA repair and oxidative stress. In this study, we sequenced our lab’s stock strain of *D. radiodurans* R1 ATCC 13939, referred to as ATCC 13939K, and compared it with the published ATCC BAA-816 genome to pinpoint genetic variations.

## 2 Results

### 2.1 Genomic features of *Deinococcus radiodurans* ATCC 13939K

The genomic characteristics of ATCC 13939K were analyzed in comparison with the ATCC BAA-816 strain and three additional *D. radiodurans* R1 genome sequences available in the National Center for Biotechnology Information’s (NCBI) Genome database. These sequences, referred to as R1-2016 (Hua and Hua, 2016), ATCC 13939E (Repar et al., 2021), and ATCC 13939O (Eugénie et al., 2021), were included to provide a comprehensive comparison of genomic attributes across strains (Table 1). Each strain encompasses two circular chromosomes and two plasmids, pMP and pCP. The aggregate genome length of ATCC 13939K stands at 3,285,071 bp, accompanied by a G + C content of 66.65%. Bioinformatic predictions have identified a sum of 3,150 protein-coding sequences (CDSs), in addition to 50 tRNA genes and nine rRNA genes (with three copies each of 5S, 16S, and 23S) within its genome (Table 1). The genome magnitude of ATCC 13939K aligns closely with that of BAA-816 (3,284,156 bp) and is roughly six kbp in excess compared to ATCC 13939E and ATCC 13939O. This disparity is ascribed mainly to variations in the length of chromosome 1 among the strains (Table 1). Prominent insertions discerned in chromosome 2, as well as the two plasmids of R1-2016 (Hua and Hua, 2016), were absent in other R1 strains (Table 1). In pairwise alignments, ATCC 13939K exhibited a 99.98% nucleotide-level identity with BAA-816 and manifested considerable synteny conservation, underscoring its collinearity and the absence of pronounced genomic structural variations.

**Table 1:** Genomic features of *D. radiodurans* R1 strains.

Features	ATCC 13939K	ATCC BAA-816^*^	R1-2016^*^	ATCC 13939E^*^	ATCC 13939O^*^
Genome size (total)	3,285,071 bp	3,284,156 bp	3,344,765 bp	3,279,598 bp	3,279,219 bp
Chromosome 1	2,650,014 bp (CP150840)	2,648,638 bp (NC_001263.1)	2,646,742 bp (NZ_CP015081.1)	2,644,543 bp (NZ_CP038663.1)	2,644,251 bp (NZ_CP068791.1)
Chromosome 2	412,190 bp (CP150841)	412,348 bp (NC_001264.1)	433,133 bp (NZ_CP015082.1)	412,189 bp (NZ_CP038664.1)	412,138 bp (NZ_CP068792.1)
Plasmid (pMP)	177,364 bp (CP150842)	177,466 bp (NC_000958.1)	203,183 bp (NZ_CP015083.1)	177,363 bp (NZ_CP038665.1)	177,322 bp (NZ_CP068793.1)
Plasmid (pCP)	45,503 bp (CP150843)	45,704 bp (NC_000959.1)	61,707 bp (NZ_CP015084.1)	45,503 bp (NZ_CP038666.1)	45,508 bp (NZ_CP068794.1)
GC content	66.65%	66.61%	66.62%	66.68%	66.68%
CDSs	3,150	3,126	3,155	3,118	3,092
tRNAs	50	50	50	50	50
rRNAs	9	9	9	9	9
Locus tag prefix	KDR	DR	A2G07	E5E91	DRO

* Genome sequences were retrieved from the NCBI Genome database.

### 2.2 Sequence discrepancy analysis between ATCC 13939K and BAA-816

In the genomic comparison between BAA-816 and ATCC 13939K, a total of 436 DNA sequence differences were identified, including 100 single nucleotide variations (SNVs), 278 insertions with sizes ranging from 1 to 6 base pairs (bp), and 58 short deletions (Table 2 and Supplementary Table S1). Previous studies have shown that R1-2016 and ATCC 13939E exhibit 577 and 559 genetic variations compared to BAA-816 (Hua and Hua, 2016; Repar et al., 2021). The genomic alignment of these differences with the BAA-816 genome revealed that 107 are located in intergenic regions, while the majority, 329 (75.7%), occur within gene regions, including pseudogenes and the 23S rRNA genes (Table 2 and Supplementary Table S1). These variations are observed across 259 distinct genes, with several genes harboring multiple differences. For instance, gene *DR*_*1922*, encoding exonuclease SbcC, presented with 4 SNVs and 5 insertional events (Supplementary Table S1). Among the 76 SNVs detected in CDSs, 21 were synonymous substitutions, while 53 led to amino acid (aa) changes. Notably, 2 SNVs lead to the elimination of stop codons in genes *DR*_*1333* and *DR*_*2250* (Supplementary Table S1). Furthermore, 241 insertion or deletion (InDel) events were identified, resulting in frameshifts in 164 CDSs and alterations in the reading frames of 46 pseudogenes (Supplementary Table S1).

**Table 2:** Number of SNV and InDel events.

	Genetic variation type (Gene region/Intergenic region^*^)			
	Insertion	Deletion	Substitution	Total
Chromosome 1	159/59	37/11	69/14	265/84
Chromosome 2	29/3	3/1	7/0	39/4
pMP	5/2	1/1	5/0	11/3
pCP	10/11	1/3	3/2	14/16
Total	203/75	42/16	84/16	329/107

* Sequence differences identified from ATCC 13939K were mapped to the genome of BAA-816.

### 2.3 Revising the gene annotation of *Deinococcus radiodurans*

Predominantly, InDel events lead to modifications in the length of CDSs or cause the fusion of genes. In the latter case, two or three adjacent genes may merge to form a singular CDS. For a comparative analysis of alterations in the CDSs, we reconfigured the initiation positions of each chromosome and plasmid in ATCC 13939K to coincide with those of BAA-816. Additionally, the designation of the predicted CDSs prefixed with ‘KDR’ was synchronized with the inaugural annotation of the BAA-816 genes. Within the BAA-816 genome, the first gene of chromosome 1, designated *DR*_*0001*, is characterized to encode the DNA polymerase III β clamp (DnaN) consisting of 393 aa, as per the initial annotation provided by the authors (GenBank genome AE000513.1). However, this CDS is omitted in the annotation crafted by NCBI (NC_001263.1) (Supplementary Table S2). This absence suggests that the protein homology in particular segments might be insufficient to validate protein-coding designations robustly (Tatusova et al., 2016). In the ATCC 13939K genome, the *dnaN* gene is newly annotated and assigned the locus tag KDR_0001. This gene spans 1,086 nucleotides and encodes a β-clamp of 361 aa (Supplementary Table S2), a consequence of a 1-bp deletion corresponding to guanine (G) at the 1,037th position in *DR*_*0001* (Supplementary Table S1). This observation is consistent with a previous study (Niiranen et al., 2015).

A comparative genomic analysis among *Deinococcus* species revealed several annotation inaccuracies in the foundational genomes of BAA-816 (AE000513.1, AE001825.1, AE001826.1, and AE00001827.1). Many of these misannotations were rectified in the reannotated genomes (NC_001263.1, NC_001264.1, NC_000958.1, and NC_000959.1) using the annotation protocols from NCBI. More information on this can be found at https://ncbi.nlm.nih.gov/refseq/about/prokaryotes/reannotation/. An illustrative example is *DR*_*0003*. When its orientation is reversed, this gene encodes the DNA damage response protein DdrC (de Groot et al., 2009). Within the reference sequence NC_001263.1, DdrC is appropriately annotated as DR_RS00015 and corresponds to KDR_0003r from ATCC 13939K (Supplementary Table S2). Consequently, the features of the predicted CDSs from ATCC 13939K were also juxtaposed with those in the NCBI-curated annotation (Supplementary Table S2).

Upon examining the CDS across the ATCC 13939 strains, we observed that 2,557 CDSs possess the same length. In addition, 2,629 ATCC 13939K CDSs align with those in ATCC 13939E, while 2,584 match ATCC 13939O (Supplementary Table S3). Within the ATCC 13939K genome, the second gene on chromosome 1, labeled *KDR*_*0002*, is annotated as encoding a DnaA protein with a length of 454 aa. In contrast, the corresponding DnaA proteins in ATCC 13939E and ATCC 13939O are annotated as having 466 aa (Supplementary Table S3). This discrepancy arises from different annotation procedures, as the gene exhibits no sequence differences (Supplementary Table S1). Considering the three CDSs, DR_RS00010, E5E91_RS00010, and DRO_RS00010, annotated by NCBI share consistency (Supplementary Tables S2, S3), there is a possibility that the length of KDR_0002 might undergo revision in future NCBI annotations. In addition, among the newly identified CDSs in ATCC 13939K, several with a length of less than 50 aa, such as KDR_0131n, 0333n, and 0347n, are absent in other 13,939 strains (Supplementary Table S3), indicating that NCBI may exclude these short CDSs in their annotation processes. To better understand the genetic variations across the sequenced R1 strains, we juxtaposed the ATCC 13939K genes that exhibit sequence differences (Supplementary Table S1) against their counterparts annotated in R1-2016, ATCC 13939E, and ATCC 13939O (Supplementary Table S4).

#### 2.3.1 DNA repair proteins

In *D. radiodurans*, one of the most critical stress response mechanisms is the DNA repair process. Numerous research articles published in the last few decades have focused on the composition of DNA repair systems and found discrepancies in the BAA-816 genome sequence. For example, *DR_0099* is predicted to code for a single-stranded DNA-binding (SSB) protein consisting of 143 aa in strain BAA-816 (Supplementary Table S2). However, in ATCC 13939, a continuous 906-bp ORF that includes *DR*_*0099* and *DR*_*0100* has been identified, encoding a larger deinococcal SSB protein (Eggington et al., 2004), which matches the SSB proteins observed in ATCC 13939K and other R1 strains examined in this study (Supplementary Table S4).

In the ATCC 13939K strain, the genes *DR_1258* and *DR_1259*, initially believed to code for the SNF2/Rad54 helicase-related protein and SNF2/Rad54 helicase, respectively, were identified as a single ORF (*KDR_1258m*) encoding Snf2 intein (Supplementary Table S4). The existence and splicing activity of this Snf2 intein were empirically validated (Southworth and Perler, 2002). Frameshifts were identified in several DNA replication and repair genes, such as *dnaN* (*DR_0001*), *mutS1* (*DR_1039*), and *recJ* (*DR_1126*) in strain ATCC 13939K (Supplementary Table S1), aligning with previous research (Mennecier et al., 2004; Bentchikou et al., 2010; Niiranen et al., 2015). These CDSs, absent in the BAA-816 genome NC_001263.1, have been restored in ATCC 13939K and other R1 strains (Supplementary Table S4).

**Figure 1:**
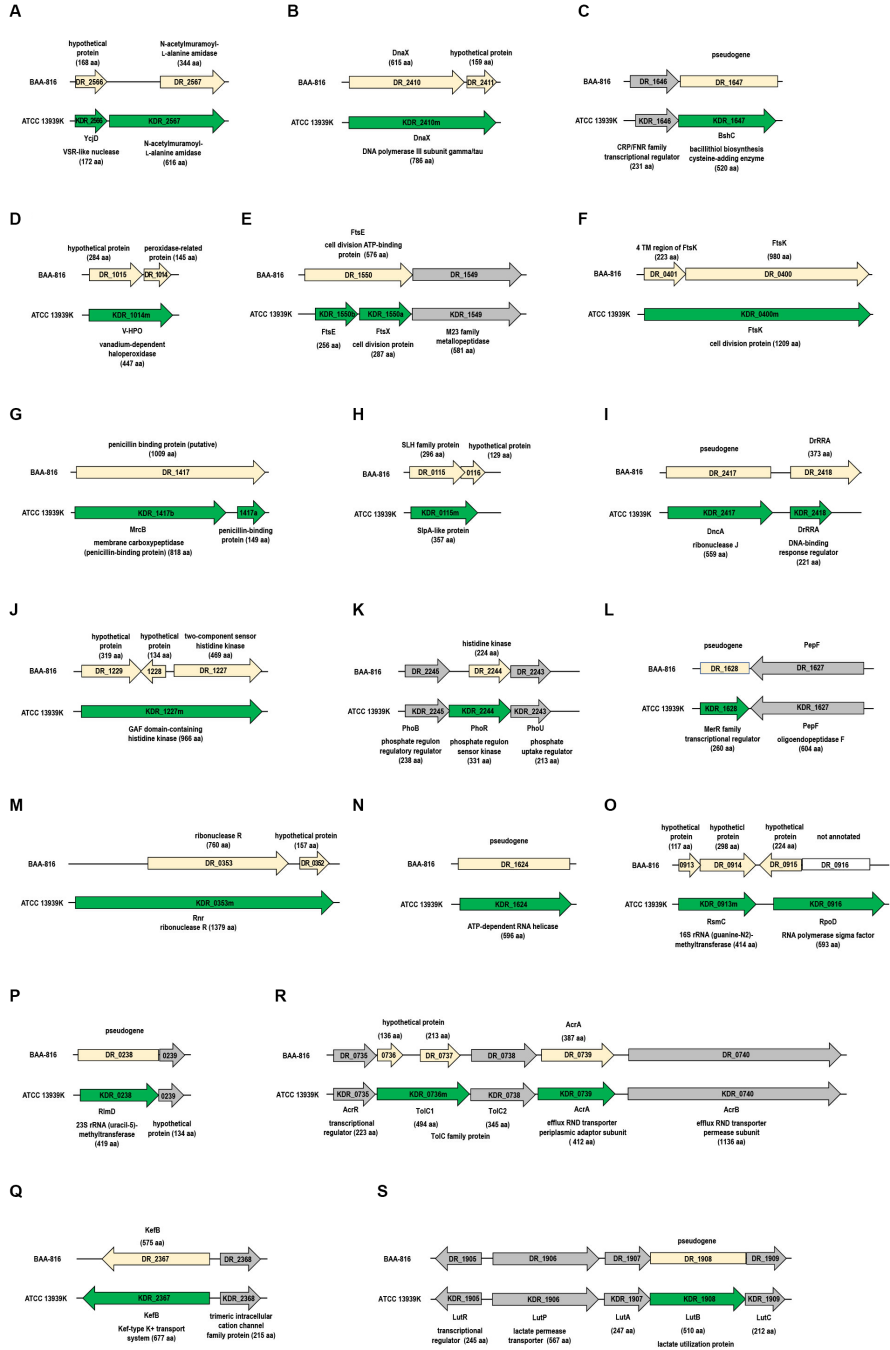
Differences in gene configurations between the BAA-816 and ATCC 13939K strains. Light yellow arrows represent genes from the BAA-816 strain, while genes from the ATCC 13939K strain are depicted with green arrows. Genes are categorized by their protein functions: **(A,B)** DNA repair proteins, **(C,D)** antioxidant proteins, **(E,F)** cell division proteins, **(G,H)** cell wall proteins, **(I–K)** two-component regulatory systems, **(L,M)** transcriptional regulators, **(N–P)** RNA metabolism proteins, and (Q to S) transporters. Conserved genes that are present in both strains are indicated with gray arrows. The direction of transcription for each gene is marked with arrowheads. Each gene is labeled with its locus tag, corresponding to the description of its associated protein.

The protein DR_2566, which includes the domain of unknown function (DUF) 2,726 (Supplementary Table S2), is presumed to be a very short patch repair (VSR)-like nuclease (Slade and Radman, 2011; Steczkiewicz et al., 2012). While DR_2566 and KDR_2566 have comparable lengths of 168 aa and 172 aa, respectively (Figure 1A and Supplementary Table S4), they show variations in the 1 to 60 aa residue region at their N-termini (Supplementary Figure S1). An examination of the *KDR*_*2566* nucleotide sequence indicates the presence of an additional cytosine (C) at position 182, leading to a frameshift in the N-terminal region (Supplementary Figure S1 and Supplementary Table S1).

The DNA polymerase III holoenzyme is composed of two dimerized β subunits, a core Pol III dimer, and a unique γ complex (consisting of γ, τ, δ, δ’, χ, ψ) that assists in loading the β processivity clamp to the DNA template. Both γ and τ proteins are encoded from the *dnaX* gene (Hejna and Moses, 2009). While the *dnaX* ORF aligns with the τ protein’s size, the shorter γ protein, which aligns with τ’s N-terminal fragment, is generated through programmed translational frameshifting (Farabaugh, 1996). Although *DR*_*2410* was predicted to produce both proteins, a significant size discrepancy was observed in τ (Makarova et al., 2001). Our observations indicate that strain ATCC 13939K lacks the thymine (T) at position 1,825 in *DR_2410* (Supplementary Figure S2). As a result, *DR_2410* and *DR_2411* merge into a unified ORF in ATCC 13939K, termed *KDR_2410m* (Figure 1B). The length of DnaX in other R1 strains is also 786 aa (Supplementary Table S4), suggesting a need for further experimental validation to determine the synthesis and accurate size of γ and τ.

#### 2.3.2 Antioxidant proteins

In *D. radiodurans*, the thiol known as bacillithiol (BSH) plays a role in neutralizing hydrogen peroxide (H_2_O_2_) in collaboration with the unique bacilliredoxin AbxC (Jeong et al., 2021). The enzyme BshC, which is integral to the final stage of BSH synthesis by adding cysteine, is commonly observed to have a length exceeding 500 aa in BSH-producing bacteria (Gaballa et al., 2010). In the *KDR*_*1647* DNA sequence, an extra C at the 954th position leads to coding for a 520-aa BshC (Figure 1C and Supplementary Figure S3). In contrast, *DR*_*1647* is annotated as a pseudogene (Supplementary Table S2).

Vanadium-dependent haloperoxidases (V-HPOs) are part of a group of non-heme enzymes that utilize peroxide alongside manganese catalases and thiol peroxidases, such as peroxiredoxins and glutathione peroxidases (Bernroitner et al., 2009). V-HPOs facilitate the oxidation of halides (including I^−^, Br^−^, and Cl^−^) when H_2_O_2_ is present (Leblanc et al., 2015). In the BAA-816 strain, the genes *DR*_*1015* and *DR*_*1014*, coding for a hypothetical protein and a peroxidase-related protein, respectively, are separate (Figure 1D). But, in the ATCC 13939K strain, these genes combine into a single 1,344 bp gene, *KDR*_*1014m*, encoding V-HPO. This combination is due to G found at the 778th position (Figure 1D and Supplementary Figure S4).

#### 2.3.3 Ddr and Ppr proteins

The Ddr (DNA damage response) and Ppr (pleiotropic protein promoting DNA repair) proteins in *D. radiodurans* are crucial for the bacterium’s remarkable ability to withstand and repair extreme DNA damage (Lim et al., 2019). Similarly to *ddrC*, *ddrH* is transcribed from the reverse strand at locus DR_0438 (de Groot et al., 2009). The *ddrH* gene is correctly annotated as *KDR_0438r* in ATCC 13939K (Supplementary Table S5). In the BAA-816 strain, *DR_0997* (*ddrI*) is predicted to encode a 260-aa cyclic AMP receptor protein (CRP), which acts as a global transcriptional regulator. Deletion of G at position 543 in *DR_0997* leads to a truncated version of the DdrI protein in ATCC 13939 (Yang et al., 2016). This deletion is noted in *KDR_0997*, resulting in a DdrI protein of 203 aa in length (Supplementary Tables S1, S5). The insertion of G into *DR_1440* restored the complete ORF for *KDR_1440*, along with its functional protein form (Supplementary Tables S1, S5). KDR_1440, known as DdrM, potentially functions as an iron efflux protein, contributing to iron homeostasis and enhancing resistance to oxidative stress in *D. radiodurans* (Dai et al., 2018).

PprI is a protease that activates DNA repair genes by cleaving DdrO. This trans-acting repressor binds to RDRM (radiation/desiccation response motif) sequences in gene promoters (Lim et al., 2019). These RDRM sequences, acting as cis-regulatory elements, are integral to the radiation desiccation response (RDR) genes in *D. radiodurans* (Eugénie et al., 2021). Frameshifts were detected in several RDR proteins, including SSB, MutS1, HelD, DR_C0017, and DR_C0023 (Supplementary Tables S1, S5).

#### 2.3.4 Cell division proteins

The cell division proteins of *D. radiodurans*, FtsA (DR_0630) and FtsZ (DR_0631), have been examined through both *in vitro* and *in vivo* studies (Modi and Misra, 2014; Maurya et al., 2018). For other Fts proteins like FtsE (DR_1550), FtsW (DR_2497), and FtsQ (DR_0629), their interactions with chromosome partitioning Par proteins have been investigated (Maurya et al., 2016). FtsE acts as the cytoplasmic ATP-binding component, while FtsX is the membrane-bound counterpart. These two proteins form a membrane-associated complex resembling an ATP-binding cassette (ABC)-type transporter. The *ftsE* and *ftsX* genes are located side-by-side in an operon (Pichoff et al., 2019). In the BAA-816 strain, DR_1550 is identified as a 576-aa FtsE. But, in the ATCC 13939K strain, this is divided into FtsE (KDR_1550b) and FtsX (KDR_1550a) (Figure 1E). This FtsEX complex plays a role in controlling the activities of the periplasmic peptidoglycan (PG) hydrolases (Pichoff et al., 2019). Notably, in the ATCC 13939K strain, the *ftsE* and *ftsX* genes are co-located with the *KDR_1549* gene (Figure 1E), which encodes for enzymes from the M23 metallopeptidase family, commonly recognized for their PG hydrolase activity (Razew et al., 2022).

In *Escherichia coli* (*E. coli*), three N-acetylmuramoyl-l-alanine amidases, AmiA, AmiB, and AmiC, primarily facilitate PG hydrolysis (Pichoff et al., 2019). Putative amidase proteins, including DR_1387, DR_1632, DR_2394, DR_2567, and DR_C0013, have been identified in the BAA-816 genome through KEGG mapping. The G to C change, positioned 25 nucleotides upstream of the GTG start codon for DR_2567, results in a long ORF termed KDR_2567 in strain ATCC 13939K (Supplementary Figure S5). This modified ORF encodes a protein of 616 aa, in contrast to the 344 aa of the DR_2567 protein predicted from the BAA-8166 genome sequence, featuring an extension of 272 aa at its N-terminal end (Figure 1A). FtsK, involved in cell division and chromosome segregation, possesses transmembrane (TM) domains at its N-terminus (Bisicchia et al., 2013). In the strain ATCC 13939K, the proteins DR_0400 (annotated as FtsK) and DR_0401 (representing the 4TM domains of FtsK) are consolidated into a single protein, KDR_0400m (Figure 1F), corroborating a recent finding (Mishra et al., 2022).

#### 2.3.5 Cell wall proteins

The *mrcB* gene, corresponding to locus tag DR_1417 in BAA-816, encodes the penicillin-binding protein 1b (PBP1b), which is crucial for preserving PG integrity and structure (Vigouroux et al., 2020). In BAA-816, this protein is 1,009 aa long, whereas it spans 807 to 818 aa in other R1 strains (Figure 1G and Supplementary Table S4). SlpA (DR_2577) is a primary S-layer protein responsible for sustaining the integrity of the *D. radiodurans* cell envelope (Farci et al., 2015). Other proteins like DR_0383, DR_1115, DR_1124, and DR_1185 are also identified as potential S-layer proteins (Makarova et al., 2001). Notably, in the ATCC 13939K strain, *DR*_*0115* and *DR*_*0116*, which are separate in BAA-816, merge to form a singular gene, *KDR*_*0115m* (Figure 1H), which encodes for a SlpA-like protein (von Kügelgen et al., 2022). *KDR_2572m*, a fusion of *DR_2572* and DR_2573, encodes a bactofilin with potential cytoskeletal functions (Supplementary Table S4). However, no reported studies have explored the functional role of bactofilins in *D. radiodurans.*

#### 2.3.6 Two-component regulatory systems

The two-component systems (TCSs), which comprise a sensor histidine kinase (HK) and a cytoplasmic response regulator (RR), are pivotal in the radioresistance of *Deinococcus species* (Lim et al., 2019). DrRRA is a novel RR essential for the radioresistance of *D. radiodurans*. Mutations in this gene reduce γ-radiation resistance and induce widespread transcriptional changes in numerous genes, predominantly those associated with DNA repair (Wang et al., 2008). Insertion of a G at the 647^th^ position results in a frameshift in the *DrRRA* gene of ATCC 13939K (Supplementary Table S1). Consequently, the DrRRA protein (KDR_2418) is shortened to 221 aa, compared to the 373 aa previously described for BAA_816 (Figure 1I). Expression analysis confirms that the actual size of DrRRA is 221 aa (Liu et al., 2012). KDR_1227m features two consecutive GAF domains in its N-terminal region and an HK domain at the C-terminus. This configuration appears as a merged product of DR_1227, DR_1228, and DR_1229 (Figure 1J).

In bacterial cells, the PhoBR TCS, comprising PhoB (RR) and PhoR (HK), triggers the transcription of genes that encode the PstSCAB system. This ABC transporter system is essential for the high-affinity uptake of phosphate ions, particularly under phosphate-deprived conditions (Santos-Beneit, 2015). In *D. radiodurans*, the PstSCAB complex is encoded by the genes *DR_A0157* through *DR_A0160* (Supplementary Table S2). In ATCC 13939K, a 996-bp ORF spans *DR*_*2244* and its upstream region, labeled as *KDR_2244*, encoding a 331 aa PhoR protein (Figure 1K). This ORF contains an additional C at position 2,238,476, situated 150 nucleotides upstream of the GTG translation initiation codon of *DR*_*2244* (Supplementary Table S1). In other R1 strains, the predicted size of this protein is 305 aa (Supplementary Table S4). Mutations in *phoR* have been observed to increase γ-radiation sensitivity (Im et al., 2013). Furthermore, when exposed to H_2_O_2_, *D. radiodurans* accumulates polyphosphate granules (Dai et al., 2021), suggesting a role for the PhoBR TCS in its oxidative stress response.

#### 2.3.7 Transcriptional regulators

PerR, a homolog of the Fur (ferric uptake repressor), acts as a transcriptional regulator in response to peroxide (Lim et al., 2019). A Fur homolog, designated DrPerR, has been newly annotated in ATCC 13939 (Liu et al., 2014). The *DrPerR* gene sequence (GeneBank accession number KJ817356) features a G insertion at position 1:2,340,150 compared to the BAA-816 genome. This insertion creates an ORF that encodes an 80 aa DrPer protein (Liu et al., 2014). The same G insertion is found in ATCC 13939K (Supplementary Table S1), leading to the assignment of the locus tag KDR_2341n to DrPerR (Supplementary Table S2).

The MerR and SmtB/ArsR families represent two general classes of transcriptional regulatory proteins that play crucial roles in heavy metal stress response (Busenlehner et al., 2003). In the genome AE000513.1, *DR*_*1628* is a pseudogene. However, in NC_001263.1, it is divided into two genes: *DR*_*RS08320*, which codes for the MerR-type helix-turn-helix (HTH) domain, and *DR*_*RS16830*, encoding the TipA_S_ antibiotic-recognition domain (Supplementary Table S2). In the ATCC 13939K strain, a nucleotide insertion at position 1,651,397 disrupted the stop codon of *DR*_*RS08320* (Supplementary Tables S1, S2), leading to the fusion of these separate genes under the tag KDR_1628, which represents a MerR family transcriptional regulator (Figure 1L).

The merged gene from *DR*_*0233* and *DR*_*0234* produces the ArsR transcriptional regulator, denoted as KDR_0233m (Supplementary Table S4). The N-terminal region of KDR_0233m includes a DNA-binding HTH domain typical of the ArsR family, while the C-terminus encompasses DUF2087 (Supplementary Table S2). A further experimental investigation is required to elucidate the functions of these newly identified full-length proteins.

#### 2.3.8 Ribonucleases

Ribonucleases (RNases) are a set of enzymes involved in all aspects of RNA metabolism. Specifically, RNase J is distinguished by its capability to serve as both an endonuclease and a processive 5′ exonuclease, crucial for RNA processing and breakdown (Richards and Belasco, 2011). While the *DR_2417* ORF is labeled as a pseudogene, *KDR*_*2417* is recognized to encode the functional RNase J (Figure 1I and Supplementary Table S2) due to a missing adenine (A) at the 995^th^ position (Supplementary Table S1). Sequencing of the PCR product amplified from ATCC 13939 confirmed the absence of frameshift (Das and Misra, 2012).

RNase II and RNase R belong to the RNR (ribonucleotide reductase) superfamily, characterized by nonspecific, 3′ to 5′ processive exoribonuclease activity. DR_0020 is classified as an RNase II-type enzyme (Schmier et al., 2012). In the BAA-816 genome, the *DR*_*0353* gene encodes an RNase R protein of 760 aa (Supplementary Table S2). In contrast, ATCC 13939K features a single ORF, *KDR_0353m*, extending across *DR_0352*, *DR_0353*, and the upstream region, which produces a 1,379 aa RNase R protein (Figure 1M). Typically, deinococcal RNase R proteins range in size from 1,000 to 1,500 aa (data not shown).

#### 2.3.9 RNA metabolism proteins

RNA helicases, which modify RNA secondary structures and RNA-protein interactions, play essential roles in RNA metabolism (Owttrim, 2013). Identified within the DEAD-box protein family is a presumptive RNA helicase, KDR_1624, which manifests as a full-length protein comprising 596 aa (Figure 1N). A two-nucleotide deletion in *DR*_*1624*, previously annotated as a pseudogene in BAA-816 (Supplementary Table S1), restored its ORF.

A two-nucleotide deletion in DR_1624, previously annotated as a pseudogene in BAA-816, corrected the frameshift error (Supplementary Table S1).

S-adenosylmethionine-dependent methyltransferases (MTases) play a role in RNA post-transcriptional modifications by adding a methyl group to ribosomal RNA (rRNA) nucleotides (Mosquera-Rendón et al., 2014). One such MTase, RsmC, modifies G to 2-methylguanosine (m^2^G) at the 1,207 position of 16S rRNA and can function as an RNA chaperone protein, facilitating ribosome assembly (Keshav et al., 2020). In ATCC 13939K, *KDR_0913m*, a unified ORF of *DR_0913* and *DR_0914*, is identified as RsmC (Figure 1O). RlmD catalyzes the formation of 5-methyl-uridine at position 1939 (m^5^U 1939) in 23S rRNA, a function widely observed in bacteria and eukaryotes (McCown et al., 2020). While *KDR*_*0238* encodes this protein in ATCC 13939K, *DR*_*0238* is classified as a pseudogene in BAA-816 (Figure 1P).

#### 2.3.10 Transporters

The translocation and assembly module (TAM) plays a role in the transporting and secretion of outer membrane proteins. It has been reported that the three ORFs, *DR_1460*, *DR1461*, and *DR_1462*, merge to form a single ORF that encodes a 4,002 aa long TamB homolog (Yu et al., 2017). Comparison between BAA-816 and ATCC 13939K reveals five individual G insertions and one SNV in the KDR_1460m locus (Supplementary Tables S1, S2), aligning with the previous report (Yu et al., 2017). *DR*_*2367* encodes the potassium-efflux system protein KefB, which is 575 aa long. In ATCC 13939K, a G insertion at *DR*_*2367*’s stop codon creates a frameshift, extending the KefB protein (KDR_2367) by 100 aa, with an additional G insertion at position 2,365,232 (Figure 1Q and Supplementary Table S1). The resulting 677-aa KefB protein includes a Zn-finger-like domain at its C-terminus.

Resistance-nodulation-cell division (RND) transporters are drug efflux pumps known for removing various toxic substances, including antibiotics. Initially believed to be unique to gram-negative (GN) bacteria, genes that encode proteins with the structural hallmarks of RND systems have been identified in gram-positive (GP) organisms like *Corynebacterium glutamicum* and *Bacillus subtilis* (Schindler and Kaatz, 2016). A well-known example is the AcrAB-TolC system in *E. coli*. The tripartite RND systems are typically composed of the transmembrane AcrB protein (1,049 aa), the periplasmic AcrA protein (397 aa), and the outer membrane TolC protein (493 aa) (Jang, 2023). The local repressor AcrR is also upstream of the *acrAB* operon (Alvarez-Ortega et al., 2013). In ATCC 13939K, a unified ORF combining *DR_0736* and *DR_0737*, designated as *KDR_0736m*, encodes TolC1 (494 aa) (Supplementary Table S4). The *KDR*_*0735*-*KDR*_*0740* operon, which codes for AcrR, AcrA, AcrB, and TolC (Figure 1R), suggests that the RND complex in *D. radiodurans* is operational.

In *D. radiodurans*, fructose is the preferred carbohydrate source (Venkateswaran et al., 2000). The bacterial phosphoenolpyruvate phosphotransferase system (PTS), responsible for carbohydrate transport and phosphorylation, includes cytoplasmic energy-coupling proteins such as enzyme I (EI) and HPr, along with sugar-specific enzyme II (EII) complexes (Comas et al., 2008). In *Pseudomonas putida*, the *fruR*-*fruBKA* operon is involved in fructose uptake, where *fruB* codes for a unique multi-phosphoryl transfer protein that integrates EIIA^Fru^-HPr-EI domains (Chavarría et al., 2016). Upon resequencing, a complete coding sequence for FruB (KDR_B0075) was identified in ATCC-13939K (Supplementary Tables S1, S4). Additionally, the entire *fruR*-*fruBKA* operon is preserved in the ATCC 13939 strains (Supplementary Table S3).

In *B. subtilis*, lactate utilization, specifically l-lactate conversion to pyruvate, is facilitated by the *lutABC* operon, which encodes three iron–sulfur-containing proteins. Typically, this conserved operon is situated alongside the *lutR* and *lutP* genes responsible for coding a transcriptional regulator and lactate permease, respectively (Chai et al., 2009). However, in the case of *DR*_*1908*, which aligns with *lutB*, it’s noted as a pseudogene, with only a partial LutB protein (DR_RS09770) present in the NCBI-annotated genome NC_001263.1 (Supplementary Table S2). A previously identified frameshift in *DR*_*1908* (Hwang et al., 2013) has been corrected by a C insertion at position 1,928,139 in ATCC-13939K, restoring the ORF (Figure 1S and Supplementary Table S1). It is worth noting that *D. radiodurans* can use lactate as its sole carbon source (Venkateswaran et al., 2000).

### 2.4 Gene gain and loss

Gene gain and loss often happen through the addition and removal of various-sized genome segments, frequently involving mobile genetic elements (MGEs) (Iranzo et al., 2019). Intercellular MGEs like plasmids and phages facilitate DNA transfer between bacterial cells. On the other hand, DNA movement within cells is primarily mediated by specific MGEs such as transposons (Tns) and insertion sequences (ISs). IS elements represent the most basic type of Tn, containing only essential genes for their transposition process (Bennett, 2004). Notably, IS elements are more abundant in *D. radiodurans* than *E. coli* and *B. subtilis*, which serve as model organisms for GN and GP bacteria (Makarova et al., 2001).

**Figure 2:**
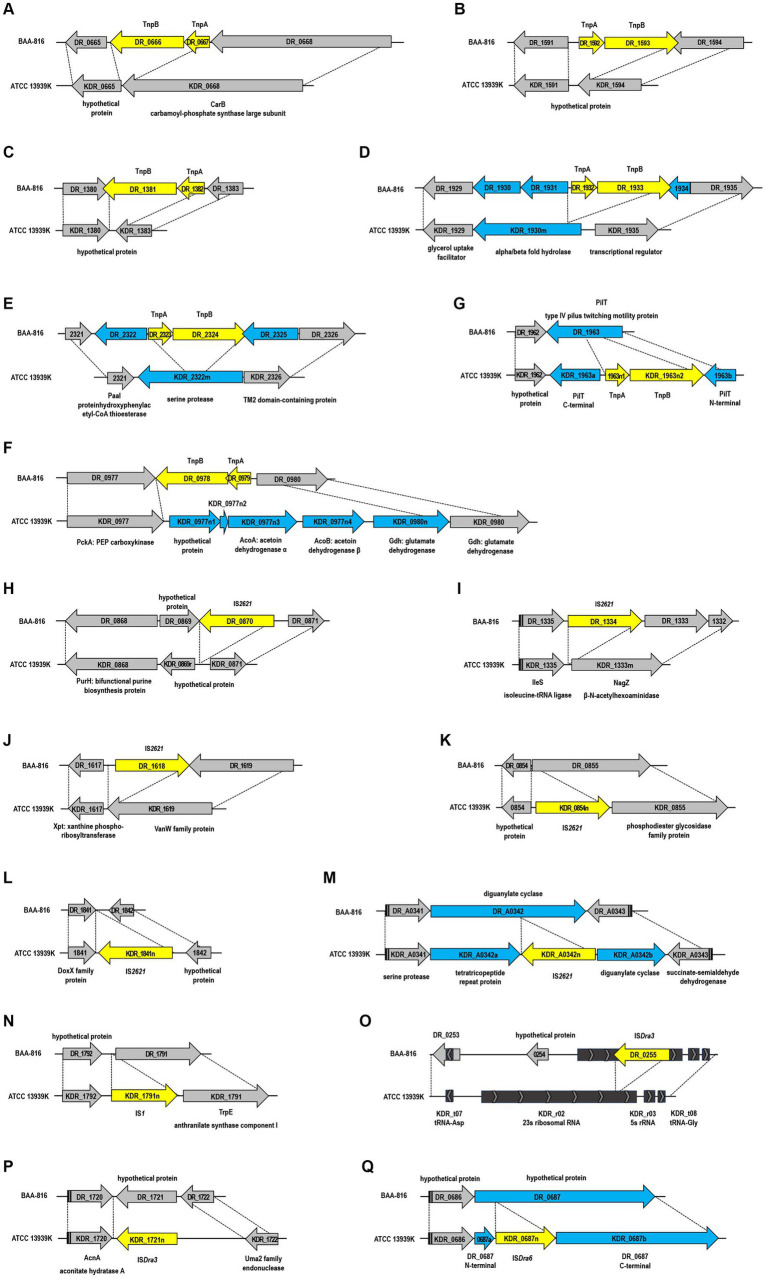
Structural genomic variations mediated by insertion sequences (IS) between the BAA-816 and ATCC 13939K strains. The IS elements are represented by yellow arrows and are categorized as follows: **(A–F)** IS*Dra2*, **(H–M)** IS*2621*, **(N)** IS*1*, **(O,P)** IS*Dra3*, and **(Q)** IS*Dra6*. Genes that are either restored, disrupted, or newly identified by the transposition of IS elements are denoted by blue arrows. Genes that are conserved across both strains are indicated by gray arrows. Dashed lines delineate homologous regions shared between the genomes of the two strains. Each gene is labeled with its locus tag.

In *D. radiodurans*, the IS*Dra2* element, originally termed IS*8301* and belonging to the IS*200*/IS*605* family, comprises two genes: *tnpA* and *tnpB*. These genes code for transposase and RNA-guided DNA nuclease (Islam et al., 2003; Karvelis et al., 2021). IS*Dra2*’s transposition activity significantly increases under γ-radiation or UV radiation exposure (Mennecier et al., 2006). In the ATCC 13939 strain, IS*Dra2* exists as a single functional copy (*DR*_*1652*-*DR*_*1651*) and one inactive, degenerate copy (*DR*_*0177*-*DR*_*0178*), while BAA-816 contains seven complete and one incomplete IS*Dra2* copies (Pasternak et al., 2010). Comparative genomic analysis revealed the loss of six IS*Dra2* elements in ATCC 13939K (Figures 2A–F) and other examined 13,939 strains (Supplementary Table S3). This loss led to the reconstitution of genes disrupted by IS*Dra2* insertion. For instance, *KDR*_*1930m* and *KDR*_*2322m*, coding for α/β-hydrolase fold enzyme and serine protease were restored (Figures 2D,E). In the ATCC 13939K strain, the IS*Dra2* segment *DR*_*0979*-*DR*_*0978* was replaced by five ORFs (Figure 2F). Among these ORFs, *KDR_0977n2* is not defined in other 13939 strains (Supplementary Table S3). The enzyme acetoin dehydrogenase, produced by the genes *KDR*_*0977n3* (*acoA*) and *KDR*_*0977n4* (*acoB*), plays a critical role in the breakdown of acetoin (Xiao and Xu, 2007). Additionally, the genes *KDR*_*0980n* and *KDR*_*0980* produce glutamate dehydrogenase (Gdh), which facilitates the reversible conversion of glutamate into α-ketoglutarate and ammonia (Miñambres et al., 2000). In a unique case of gene disruption by IS*Dra2*, the segment *KDR*_*1963n1*-*KDR*_*1963n2* is integrated into the *pilT* gene, which codes for the pilus retraction ATPase (Figure 2G). This disruption is exclusive to the ATCC 13939K strain and does not occur in other 13939 strains (Supplementary Table S3). Given that the DNA translocation system in *D. radiodurans* is associated with type IV pili, mutations in the *pilT* gene lead to a decrease in the efficiency of natural transformation (Ithurbide et al., 2020). The reduced transformation efficiency was observed in the ATCC 13939K strain (Supplementary Figure S6). This disruption in the 13939K strain is a notable genomic difference that affects the strain’s phenotype.

IS*2621*, spanning 1,322 bp and enclosed by 19-bp perfect inverted terminal repeats, represents another transpositionally active IS in *D. radiodurans* (Narumi et al., 1997; Mennecier et al., 2006). The BAA-816 strain originally contained five copies of IS*2621*, identified as *DR*_*0870*, *DR*_*1,334*, *DR*_*1618*, *DR*_*2222*, and *DR_B0059* (Long et al., 2015). However, in the ATCC 13939K strain, three of these copies were lost (Figures 2H–J), aligning with findings from previous research (Long et al., 2015). Additionally, ATCC 13939K acquired three new IS*2621* copies (Figures 2K–M). Among these, two copies, *KDR_0854n* and *KDR_1841n*, were exclusively found in ATCC 13939K, while the third copy, *KDR_A0342n*, was present in other 13939 strains (Supplementary Table S3). The occurrence of IS elements, specifically in ATCC 13939K, including *KDR*_*0854n*, *KDR*_*1841n*, and *KDR_1963n1*-*1963n2*, was confirmed through PCR assay (Supplementary Figure S7). Cyclic-di-GMP (c-di-GMP) acts as a crucial second messenger in the signal transduction pathways of many bacteria, regulating various cellular processes. The cellular levels of c-di-GMP are controlled by a balance between its production, catalyzed by diguanylate cyclases that contain GGDEF domains, and its breakdown, mediated by phosphodiesterases that possess either EAL or HD-GYP domains (Petchiappan et al., 2020). DR_A0342 is notable for harboring both HD-GYP and GGDEF domains within its C-terminal region, illustrating its role in c-di-GMP homeostasis (Galperin et al., 2001; Pei and Grishin, 2001). The insertion of IS*2621* into DR_A0342 splits it into two parts, KDR_A0342a and KDR_A0342b (Figure 2M). Importantly, KDR_A0342b retains the intact domains (data not shown), suggesting its potential functionality in regulating c-di-GMP levels.

In the genome NC_001263.1, *DR*_*RS16465* is identified as IS*1*, equivalent to *KDR*_*0835r* in the ATCC 13939K strain (Supplementary Table S2). Additionally, an extra copy of IS*1* has been inserted in both ATCC 13939K (Figure 2N) and other 13939 strains (Supplementary Table S3). IS*Dra3*, IS*Dra4*, and IS*Dra6* have been classified as members of the IS*630* family in the ISFinder database.^1^ These elements are characterized by sequences containing stretches of A residues. For instance, IS*Dra3*, which includes *DR_0255*, *DR_B0139*, and *DR_C0004*, features a run of nine As, and IS*Dra6*, incorporating *DR*_*1523*, *DR*_*B0056*, and *DR_B0113*, is marked by a sequence of eight As (Baranov et al., 2005). In the BAA-816 strain, the 23S rRNA genes were not fully characterized: *rrnaA23S* (*DR_r05*) and *rrnaB23S* (*DR_r09*) had a length of only 876 nucleotides each, while *rrnaC23S* (*DR_r02*) extended to 1,943 nucleotides, a result of the insertion by IS*Dra3* element *DR_0255* (Pei et al., 2009; Supplementary Table S2). In ATCC 13939K, re-annotation and the absence of *DR*_*0255* allowed for the identification of *KDR_r02*, *KDR_r05*, and *KDR_r09*, each at 2,876 nucleotides, harmonizing the lengths of all three 23S rRNA genes (Figure 2O and Supplementary Table S2). Furthermore, *KDR_1721n* and *KDR_0687n* were found as additional insertions of IS*Dra3* and IS*Dra6*, respectively, in the ATCC 13939K strain (Figures 2P,Q), consistent with the previous report (Long et al., 2015).

**Figure 3:**
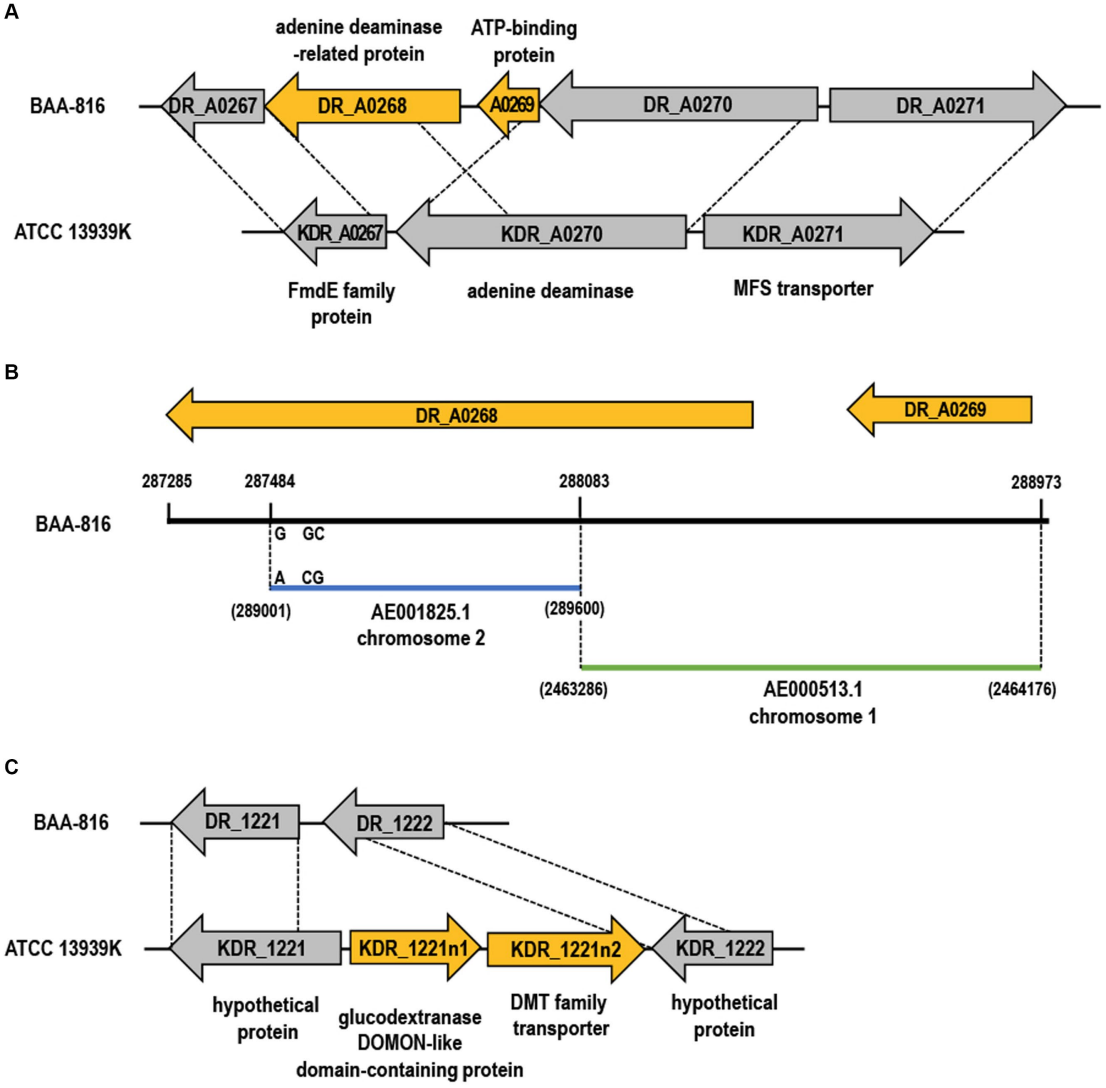
Gene gain and loss in the ATCC 13939K strain. **(A,C)** The genes lost and gained in ATCC 13939K are indicated by yellow arrows. Genes that are conserved across both strains are indicated by gray arrows. Homologous regions present in the BAA-816 strain are connected with dashed lines, and each gene is assigned a locus tag. **(B)** Chromosomal comparison of genes DR_A0268 and DR_A0269. A segment of DR_A0268 (yellow arrow) aligns nearly identically with the C-terminal end of DR_A0270 (blue bar), with a minor divergence of only three base pairs. The segment encompassing DR_A0269 (288,083–288,973) matches the region from 2,463,286 to 2,464,176 on chromosome 1 (green bar).

Gene gain or loss in bacteria can occur without MGEs due to improper recombination processes, such as homologous recombination or inaccurate non-homologous repair activities. Errors in DNA replication or the repair process, mainly when fixing aberrant replication forks, can result in the deletion or duplication of genes. Consequently, this leads to either the loss or gain of genetic material (Periwal and Scaria, 2015). The genomic comparison between 13939K and BAA-816 reveals that the genes *DR_A0268* and *DR_A0269*, present in BAA-816, are absent in the 13939K strain (Figure 3A). Examination of a 1,697 nucleotide sequence that includes *DR*_*A0268* and *DR*_*A0269* shows that a portion of *DR*_*A0268*, between coordinates 287,484 and 288,083, is nearly identical to the C-terminal sequence of *DR*_*A0270*, found between coordinates 289,001 and 289,600, with only three bases differing (Figure 3B). Additionally, the sequence spanning 288,083–288,973 on chromosome 2 aligns perfectly with the segment of chromosome 1 between coordinates 2,463,286–2,464,176 (Figure 3B). This alignment suggests that recombination between homologous sequences of *DR_A0270* and *DR_A0268* could have resulted in the loss of the *DR_A0269* region in strain ATCC 13939K. Furthermore, *DR*_*A0268* has been updated to *DR*_*RS17005* in annotations (Supplementary Table S2), and the C-terminal aa sequences of KDR_A0270 match those of DR_RS17005 (Supplementary Figure S8). In the ATCC 13939K strain, there was a notable presence of two newly inserted genes, *KDR_1221n1* and *KDR_1221n2* (Figure 3C). Proteins in the drug/metabolite transporter (DMT) superfamily are characterized by a unique structure that includes varying numbers of TM α-helices, commonly at counts of 4, 5, 9, or 10 per protein. The DMT proteins with 10 TM segments are formed by duplicating a fundamental five-segment precursor within the gene (Jack et al., 2001). Specifically, the DMT protein encoded by gene *KDR_1221n2* possesses 10 helical segments (data not shown), implying that it may have evolved through a gene duplication event.

## 3 Discussion

Identified initially as strain R1 in 1956 and cataloged as ATCC 13939, the *D. radiodurans* strain was subject to a later correction when it was determined that the sequenced genome belonged to a different strain, thereby reclassifying it as ATCC BAA-816. In this study, we sequenced the *D. radiodurans* strain ATCC 13939K and unveiled genetic differences relative to ATCC BAA-816. The comparative analysis uncovered many genetic variations, including SNVs and InDels, that substantially influence gene functions and structures. These variations induced alterations in CDS lengths, the restoration of pseudogenes, and occurrences of gene fusion and splits. Despite these genetic differences, phenotypic assays for resistance to γ-radiation, UV, H_2_O_2_, and mitomycin C showed similar survival rates between strains 13939K and BAA-816 (Supplementary Figure S9). The polymorphisms largely concur with those in other analyzed *D. radiodurans* strains (R1-2016, 13939E, and 13939O) (Supplementary Table S4). These findings suggest that many genetic discrepancies between the strains are likely attributable to sequencing errors in the BAA-816 reference genome rather than variations from different laboratory cultivation practices. This assumption is substantiated by proteomic analyses that have corrected previously identified frameshifts in the *D. radiodurans* genome (Willems et al., 2020). While resequencing of the R1 ATCC BAA-816 genome is needed for confirmation, updating the *D. radiodurans* genome sequence promises substantial insights for *Deinococcus*-focused research and broader studies into microbial resilience and DNA repair mechanisms.

In *D. radiodurans*, gene gain and loss are facilitated by IS elements. These elements can change their insertion location and copy numbers, introduce novel genetic functionalities, or alter gene activity, substantially impacting bacterial fitness (Vale et al., 2022). This dynamic reshaping of the genome is exemplified in the ATCC 13939K strain, where the IS*Dra2* element’s transposition resulted in the introduction of the genes necessary for acetoin metabolism (Figure 2F). Acetoin (3-hydroxy-2-butanone) is crucial in bacterial metabolism, acting as an essential intermediary for energy production from nutrients (Xiao and Xu, 2007). The pathway begins with α-acetolactate synthase (ALS) combining two pyruvate molecules into α-acetolactate (AL), which is converted to acetoin via α-acetolactate decarboxylase (ALD) or to diacetyl through non-enzymatic oxidative decarboxylation. Acetoin reductase (AR) then converts diacetyl to acetoin, which the acetoin dehydrogenase complex (ADC) transforms into acetyl-CoA for energy production via the tricarboxylic acid cycle (Xiao and Xu, 2007; Supplementary Figure S10).

The *ilvBN* genes encode α-acetohydroxy acid synthase (AHAS), another enzyme involved in AL formation, differing from ALS by its structure (Benson et al., 1996). ALS consists of a single approximately 60 kDa subunit. In contrast, AHAS is a two-subunit enzyme comprising a larger catalytic subunit (59–66 kDa) and a smaller regulatory subunit (9–35 kDa) (Eram et al., 2015). In the BAA-816 strain, the genes *DR_1516* and *DR_1517* are designated as *ilvB* and a pseudogene, respectively (Supplementary Table S2). However, a complete set of *ilvBN* genes is identified in the ATCC 13939K strain due to a C insertion within the sequence of *KDR_1517* (Supplementary Tables S1, S2). The loss of the IS*Dra2* segment *DR_0979*-*DR_0978* revealed the presence of ADC, encoded by the genes *acoA* (*KDR_0977n3*) and *acoB* (*KDR_0977n4*) (Figure 2F). These genes coding for AHAS and ADC are also present in other strains of ATCC 13939 (Supplementary Table S3). Moreover, ARs from *Thermococcus* (Ying and Ma, 2011) and *Mycobacterium* (Takeda et al., 2014) show about 30% similarity to the protein DR_A0005 (data not shown). Although a detailed functional analysis of DR_A0005 is necessary, *D. radiodurans* ATCC 13939 strains likely encompass the complete acetoin metabolism pathway (Supplementary Figure S10). This metabolic flexibility, alternating between acetoin production and consumption, allows the bacteria to adjust to variable environmental conditions, enhancing their growth and survivability. Acetoin is produced and excreted during the exponential growth phase to prevent cytoplasmic and extracellular over-acidification. In the absence of preferred carbon sources, as the culture transitions to the stationary phase, acetoin serves as a substrate to sustain culture density (Xiao and Xu, 2007). Hence, our findings enrich the comprehension of *D. radiodurans*’ adaptive mechanisms and pave the way for novel biotechnological applications.

The main pathways for integrating ammonia into biomolecules involve converting ammonia into glutamine (Gln) and glutamate (Glu) through three essential enzymes: glutamine synthetase (GS), glutamate synthase (also known as Gln:2-oxoglutarate aminotransferase, GOGAT), and glutamate dehydrogenase (GDH) (Reitzer, 2014). GS converts Glu into Gln, and GOGAT catalyzes the transfer of the amido group from Gln to 2-oxoglutarate (2OG), yielding two molecules of Glu. In the absence of GOGAT, GDH facilitates the direct synthesis of Glu from 2OG and ammonia. The GS-GOGAT pathway, therefore, represents a primary and widely conserved way of ammonia assimilation in bacteria (Pengpeng and Tan, 2013). In *D. radiodurans*, there are two versions of GS, identified as DR_2033 (GlnA3), which has been reannotated to DR_RS10425, and DR_0451 (GlnA1), along with GOGAT, encoded by *DR_0183* (*gltB*) and *DR_0182* (*gltD*) (Venkateswaran et al., 2000; Ghosal et al., 2005; Supplementary Table S2). An insertion and deletion of G nucleotide occurred in *DR*_*RS10425*, but this variation did not result in a frameshift (Supplementary Tables S1, S2). Instead, it caused a substitution of serine for isoleucine at position 496 in KDR_2033 (data not shown), maintaining the length of both GlnA3 proteins at 719 aa (Supplementary Table S2).

Given that the glutamate dehydrogenase (GDH)-dependent pathway for ammonia assimilation is energy-efficient and exhibits low ammonium affinity, it assumes a significant role in nitrogen metabolism, particularly in environments with abundant nitrogen (Pengpeng and Tan, 2013; Reitzer, 2014). GDH is categorized into three types based on its coenzyme specificity and role: NAD-dependent GDH2 (EC 1.4.1.2) primarily facilitates the production of 2-oxoglutarate (2OG) through glutamate (Glu) catabolism, while NADP-dependent GDH4 (EC 1.4.1.4) is crucial for Glu synthesis during ammonia assimilation. GDH3 (EC 1.4.1.3) is versatile, supporting both 2OG formation and the reverse process, and shows specificity for both NAD(P)H coenzymes (Jaspard, 2006). In *D. radiodurans* (BAA-816), GDH3 is identified as DR_0980 and GDH4 as DR_1718 in the KEGG Enzyme database. Across 21 *Deinococcus* species cataloged in KEGG, their genomes typically exhibit a pair of GDH3 enzymes arranged in tandem (data not shown). Based on their genomic positioning within *Deinococcus* species, it’s inferred that KDR_0980n and KDR_0980 represent forms of GDH3. Notably, KDR_0980 is 45 aa longer than DR_0980 due to a frameshift caused by C insertions (Supplementary Tables S1, S2). While it is known that *D. radiodurans* cannot directly assimilate ammonia (Venkateswaran et al., 2000), ATCC 13939 strains retain classic ammonia assimilation pathways, GDH and GS-GOGAT, within their genomes. The observed genetic variance between strains BAA-816 and ATCC 13939 suggests that research involving ATCC 13939 could offer alternative perspectives on the process of ammonia assimilation.

The ATCC 13939 strains uniformly exhibit deletions and insertions of IS elements, with IS*Dra2*, IS*2621*, and IS*Dra3* losing six, three, and one copies each, compared to the BAA-816 genome. Moreover, one insertion each of IS*2621*, IS*1*, IS*Dra3*, and IS*Dra6* is common among these strains. Unique to the ATCC 13939K strain, additional transpositions were observed: an increase of one and two copies for IS*Dra2* (*KDR_1963n1*-*1963n2*) and IS*2621* (*KDR_0854n* and *KDR_1841n*), respectively (Figure 2 and Supplementary Table S3). These IS element transpositions among the ATCC 13939 strains indicate evolutionary developments that set them apart from BAA-816, along with genetic differences stemming from laboratory cultivation. The estimated transposition rate of IS elements is 2.50 × 10^−3^ per genome per generation in wild-type *D. radiodurans* (Long et al., 2015), influencing strain evolution. Such transpositions can significantly alter phenotypes through genetic variations in protein-coding genes. In ATCC 13939 strains, the loss of IS*Dra2* reinstated genes coding for α/β fold hydrolase and serine protease (Figures 2D,E). The disruption of *pilT* by IS*Dra2* notably reduced transformation efficiency in strain 13939K (Supplementary Figure S6). These highlight the significant role of IS elements in microbial research, demonstrating their ability to alter both the genome and specific characteristics, including metabolic processe of certain strains.

## 4 Materials and methods

### 4.1 Bacterial strains and reagents

The *D. radiodurans* strains, ATCC BAA-816 and ATCC 13939, originated from the ATCC repository. Cultures were grown at 30°C in TGY broth (0.5% tryptone, 0.3% yeast extract, and 0.1% glucose) or on TGY plates supplemented with 1.5% Bacto-agar. The chemicals used throughout the study were purchased from Sigma-Aldrich (St. Louis, MO, USA).

### 4.2 Whole genome sequencing

From single colonies on TGY agar, the *D. radiodurans* ATCC 13939K strain was cultured in TGY broth, shaking at 200 rpm at 30°C for 18 h. Post-cultivation, the cells were centrifuged, and genomic DNA was extracted using the AccuPrep® Genomic DNA Extraction Kit (Bioneer, Korea) according to the manufacturer’s instructions. Specifically, 1 × 10^9^ cells were lysed using 180 μL of lysis buffer (20 mM Tris–HCl, 2 mM sodium EDTA, 1.2% Triton® X-100, pH 8.0), supplemented with 20 μL of lysozyme (100 mg/mL) and 10 μL of RNase A. The lysate was incubated at 37°C, followed by the addition of 20 μL of Proteinase K and 200 μL of GB Buffer, with subsequent incubation at 60°C. DNA purification was then performed using spin columns. For genome sequencing, a combination of Illumina NovaSeq 6000 and PacBio Sequel II platforms was used. DNA libraries were prepared using the TruSeq DNA Nano kit (Illumina, San Diego, CA, USA) and the SMRTbell Prep Kit 3.0 (PacBio, Menlo Park, CA, USA). The initial assembly was achieved with PacBio reads achieving 298.4× coverage, using the CANU V1.7 software (Koren et al., 2017), and was further refined using Illumina reads at 480.8× coverage through Pilon V1.21 software (Walker et al., 2014).

### 4.3 Functional annotations

The genome sequence comprises four circular DNA structures totaling 3,285,071 bp. Analysis of GC content was conducted using the ANI Calculator (Richter et al., 2016). Prokka (v1.13) was employed to define the genomic profile, including CDS, tRNA, and rRNA genes (Seemann, 2014). Functional annotation of identified proteins was aligned with COG and KEGG databases, and sequence verification was performed against NCBI databases using BLASTP and BLASTX. The nucleotide sequence of ATCC 13939K was deposited in the NCBI Nucleotide database (GenBank number, CP150840 ~ CP150843).

### 4.4 Comparative genome analysis

Raw read data from Illumina sequencing was used for SNP detection. Following filtration, the clean read sequences were aligned to the reference sequence using the Burrows-Wheeler Aligner (BWA, version 0.7.17) (Li and Durbin, 2010). After mapping, duplicate reads were removed using Sambamba (version 0.6.8) (Tarasov et al., 2015), and SAMTools were used to identify variants (Danecek et al., 2021). Amino acid changes from these variants were obtained using SnpEff (version 4.3 t) (Cingolani, 2022).

### 4.5 Transformation of *Deinococcus radiodurans*

For the preparation of competent cells, exponentially growing cells were collected via centrifugation and then resuspended in TGY medium at a concentration of 5 × 10^8^ cells/mL. The medium was supplemented with 30 mM CaCl_2_ and 10% (v/v) glycerol, after which it was quickly frozen and stored at −80°C for preservation. Two plasmids were used for the transformation assays: pRadgro with a chloramphenicol resistance marker, which replicates in *D. radiodurans*, and pKatAPH3, carrying a kanamycin resistance gene, used for targeted gene disruption (Ohba et al., 2005; Misra et al., 2006). For the pKatAPH3-based assay, the upstream and downstream regions (approximately 1 kb) of *bphP* (*DR_A0050*) were PCR amplified using the primer pairs, Up-F (5′-tatctcgagtcgcgcggcctgtt-3′)/Up-R (5′-tatgatatcgatgctgggcctcttccggga-3′) and Down-F (5′-tatggatccggcaacgggtcccggctcat-3′)/Down-R (5′-tatctcgagcgacgttgcggttgtcttcgcc-3′). The PCR products were digested with *Xho*I/*Eco*RV and *Bam*HI/*Pst*I to produce the upstream and downstream fragments and ligated into the pKatAPH3 vector. The recombinant plasmid was named pKatAPH3-BphP. For transformation, aliquots of competent cells (100 μL each) were thawed on ice and mixed with an equal TGY medium containing 30 mM CaCl_2_. Subsequently, 250 ng of either plasmid DNA was added. Following a 30 min incubation at 0°C, the mixture underwent heat treatment at 32°C for 90 min. After the heat treatment, 800 μL of TGY media were added to the mixture to aid in the recovery and expression of antibiotic resistance, followed by further incubation for 5 h. Diluted samples were then plated onto TGY plates containing the appropriate antibiotics for selection.

### 4.6 PCR (polymerase chain reaction)

PCR assays were utilized to validate the integration of specific IS elements within the genome of the ATCC 13939K strain. The dedicated primer pairs were used for the detection of each IS element: 0854F (5′-gtgggctcgcctagcatgtt-3′)/0855R (5′-gaacttctacccccaggcgg-3′) for *KDR_0854n*, 1841F (5′-atgttcggcgccatctggta-3′)/1842R (5′-cctcaacctctacgccgagg-3′) for *KDR_1841n*, and 1963F (5′-taggcgtccatcgtgaccatgc-3′)/1963R (5′-gatgtactcgatgatgaacgagcc-3′) for *KDR_1963n1* and *KDR_1963n2*. Amplification was carried out using PrimeSTAR DNA polymerase (TaKaRa Bio Inc., Japan) under the following conditions: an initial denaturation step at 94°C for 40 s, followed by 20 cycles of amplification consisting of 40 s at 94°C, 40 s at 57°C, and 3 min at 72°C. A final elongation step at 72°C for 5 min was performed. The PCR products were then subjected to agarose gel electrophoresis.

### 4.7 Cell survival assays

Overnight cultures in the stationary phase were inoculated into fresh TGY broth at a 1:100 dilution and grown to log phase (OD_600_ ≈ 1.0). These cultures were then diluted to an OD_600_ of approximately 0.1 in TGY broth and treated with varying concentrations of mitomycin C (MMC) and hydrogen peroxide (H₂O₂) for 1 h at 30°C. Post H₂O₂ treatment, cells were treated with catalase (Sigma-Aldrich, Saint Louis, CA, USA) to degrade any residual H₂O₂, followed by 10-fold serial dilutions in distilled water, and subsequent spotting onto TGY agar plates. At room temperature, γ-radiation exposure was carried out using a ^60^Co gamma irradiator (AECL, IR-79; MDS Nordion International Co. Ltd., Ottawa, Canada) at the Advanced Radiation Technology Institute, Republic of Korea. Ultraviolet C (UV-C) exposure was performed using a UV crosslinker (CX-2000, UVP LLC, Upland, CA, USA), where plates with spotted cells were subjected to UV-C radiation. Post-exposure, the TGY plates were incubated at 30°C for 2–3 days, allowing colony-forming unit (CFU) enumeration.

## Data availability statement

The datasets presented in this study can be found in online repositories. The names of the repository/repositories and accession number(s) can be found in the article/Supplementary material.

## Author contributions

SJ: Methodology, Investigation, Writing – review & editing, Writing – original draft. HS: Data curation, Conceptualization, Writing – original draft. J-HJ: Writing – review & editing, Writing – original draft, Validation, Supervision, Methodology, Formal analysis. K-WJ: Visualization, Validation, Writing – review & editing. SR: Supervision, Writing – review & editing. SL: Writing – review & editing, Writing – original draft, Supervision, Resources, Funding acquisition, Conceptualization.

## Footnotes

^1^ https://www-is.biotoul.fr/

## References

[ref1] Alvarez-Ortega C. Olivares J. Martínez J. L. (2013). RND multidrug efflux pumps: what are they good for? Front. Microbiol. 4:7. doi: 10.3389/fmicb.2013.00007 23386844 PMC3564043

[ref2] Appukuttan D. Singh H. Park S. H. Jung J. H. Jeong S. Seo H. S.. (2015). Engineering synthetic multistress tolerance in *Escherichia coli* by using a deinococcal response regulator, DR1558. Appl. Environ. Microbiol. 82, 1154–1166. doi: 10.1128/AEM.03371-15, PMID: 26655758 PMC4751837

[ref3] Baranov P. V. Hammer A. W. Zhou J. Gesteland R. F. Atkins J. F. (2005). Transcriptional slippage in bacteria: distribution in sequenced genomes and utilization in IS element gene expression. Genome Biol. 6:R25. doi: 10.1186/gb-2005-6-3-r25, PMID: 15774026 PMC1088944

[ref4] Bennett P. M. (2004). Genome plasticity: insertion sequence elements, transposons and integrons, and DNA rearrangement. Methods Mol. Biol. 266, 71–113. doi: 10.1385/1-59259-763-7:071, PMID: 15148416

[ref5] Benson K. H. Godon J. J. Renault P. Griffin H. G. Gasson M. G. (1996). Effect of ilvBN-encoded α-acetolactate synthase expression on diacetyl production in *Lactococcus lactis*. Appl. Microbiol. Biotechnol. 45, 107–111. doi: 10.1007/s002530050656

[ref6] Bentchikou E. Servant P. Coste G. Sommer S. (2010). A major role of the RecFOR pathway in DNA double-strand-break repair through ESDSA in *Deinococcus radiodurans*. PLoS Genet. 6:e1000774. doi: 10.1371/journal.pgen.1000774, PMID: 20090937 PMC2806897

[ref7] Bernroitner M. Zamocky M. Furtmüller P. G. Peschek G. A. Obinger C. (2009). Occurrence, phylogeny, structure, and function of catalases and peroxidases in cyanobacteria. J. Exp. Bot. 60, 423–440. doi: 10.1093/jxb/ern309, PMID: 19129167

[ref8] Bisicchia P. Steel B. Mariam Debela M. H. Löwe J. Sherratt D. (2013). The N-terminal membrane-spanning domain of the *Escherichia coli* DNA translocase FtsK hexamerizes at midcell. MBio 4:e00800-13. doi: 10.1128/mBio.00800-13 24302254 PMC3870252

[ref9] Brim H. McFarlan S. C. Fredrickson J. K. Minton K. W. Zhai M. Wackett L. P.. (2000). Engineering *Deinococcus radiodurans* for metal remediation in radioactive mixed waste environments. Nat. Biotechnol. 18, 85–90. doi: 10.1038/71986, PMID: 10625398

[ref10] Brooks B. W. Murray R. G. E. (1981). Nomenclature for “*Micrococcus radiodurans*” and other radiation-resistant cocci: *Deinococcaceae* fam. nov. and *Deinococcus* gen. nov., including five species. Int. J. Syst. Evol. Microbiol. 31, 353–360. doi: 10.1099/00207713-31-3-353

[ref11] Busenlehner L. S. Pennella M. A. Giedroc D. P. (2003). The SmtB/ArsR family of metalloregulatory transcriptional repressors: structural insights into prokaryotic metal resistance. FEMS Microbiol. Rev. 27, 131–143. doi: 10.1016/S0168-6445(03)00054-8 12829264

[ref12] Cao Z. Julin D. A. (2009). Characterization in vitro and in vivo of the DNA helicase encoded by *Deinococcus radiodurans* locus DR1572. DNA Repair 8, 612–619. doi: 10.1016/j.dnarep.2008.12.011, PMID: 19179120

[ref13] Chai Y. Kolter R. Losick R. (2009). A widely conserved gene cluster required for lactate utilization in *Bacillus subtilis* and its involvement in biofilm formation. J. Bacteriol. 191, 2423–2430. doi: 10.1128/JB.01464-08, PMID: 19201793 PMC2668416

[ref14] Chavarría M. Goñi-Moreno Á. de Lorenzo V. Nikel P. I. (2016). A metabolic widget adjusts the phosphoenolpyruvate-dependent fructose influx in *Pseudomonas putida*. mSystems 1:e00154-16. doi: 10.1128/mSystems.00154-16, PMID: 27933319 PMC5141268

[ref15] Cingolani P. (2022). Variant annotation and functional prediction: SnpEff. Methods Mol. Biol. 2493, 289–314. doi: 10.1007/978-1-0716-2293-3_19 35751823

[ref16] Comas I. González-Candelas F. Zúñiga M. (2008). Unraveling the evolutionary history of the phosphoryl-transfer chain of the phosphoenolpyruvate:phosphotransferase system through phylogenetic analyses and genome context. BMC Evol. Biol. 8:147. doi: 10.1186/1471-2148-8-147, PMID: 18485189 PMC2405797

[ref17] White O. Eisen J. A. Heidelberg J. F. Hickey E. K. Peterson J. D. Dodson R. J.. (2004). Corrections and Clarifications. Erratum: Genome sequence of the radioresistant bacterium Deinococcus radiodurans R1. Science 303:766. doi: 10.1126/science.303.5659.766b PMC4147723 10567266

[ref18] Cox M. M. Battista J. R. (2005). *Deinococcus radiodurans* − the consummate survivor. Nat. Rev. Microbiol. 3, 882–892. doi: 10.1038/nrmicro1264 16261171

[ref19] Dai S. Jin Y. Li T. Weng Y. Xu X. Zhang G.. (2018). DR1440 is a potential iron efflux protein involved in maintenance of iron homeostasis and resistance of *Deinococcus radiodurans* to oxidative stress. PLoS One 13:e0202287. doi: 10.1371/journal.pone.0202287, PMID: 30106993 PMC6091924

[ref20] Dai S. Xie Z. Wang B. Yu N. Zhao J. Zhou Y.. (2021). Dynamic polyphosphate metabolism coordinating with manganese ions defends against oxidative stress in the extreme bacterium *Deinococcus radiodurans*. Appl. Environ. Microbiol. 87:e02785-20. doi: 10.1128/AEM.02785-20, PMID: 33452031 PMC8091604

[ref21] Daly M. J. Gaidamakova E. K. Matrosova V. Y. Kiang J. G. Fukumoto R. Lee D. Y.. (2010). Small-molecule antioxidant proteome-shields in *Deinococcus radiodurans*. PLoS One 5:e12570. doi: 10.1371/journal.pone.0012570, PMID: 20838443 PMC2933237

[ref22] Danecek P. Bonfield J. K. Liddle J. Marshall J. Ohan V. Pollard M. O.. (2021). Twelve years of SAMtools and BCFtools. Gigascience 10:giab008. doi: 10.1093/gigascience/giab008, PMID: 33590861 PMC7931819

[ref23] Das A. D. Misra H. S. (2012). DR2417, a hypothetical protein characterized as a novel β-CASP family nuclease in radiation resistant bacterium, *Deinococcus radiodurans*. Biochim. Biophys. Acta 1820, 1052–1061. doi: 10.1016/j.bbagen.2012.03.014 22503789

[ref24] de Groot A. Dulermo R. Ortet P. Blanchard L. Guérin P. Fernandez B.. (2009). Alliance of proteomics and genomics to unravel the specificities of Sahara bacterium *Deinococcus deserti*. PLoS Genet. 5:e1000434. doi: 10.1371/journal.pgen.1000434, PMID: 19370165 PMC2669436

[ref25] Eggington J. M. Haruta N. Wood E. A. Cox M. M. (2004). The single-stranded DNA-binding protein of *Deinococcus radiodurans*. BMC Microbiol. 4:2. doi: 10.1186/1471-2180-4-2, PMID: 14718065 PMC331404

[ref26] Elbakry A. Löbrich M. (2021). Homologous recombination subpathways: a tangle to resolve. Front. Genet. 12:723847. doi: 10.3389/fgene.2021.723847, PMID: 34408777 PMC8365153

[ref27] Eram M. S. Sarafuddin B. Gong F. Ma K. (2015). Characterization of acetohydroxyacid synthase from the hyperthermophilic bacterium *Thermotoga maritima*. Biochem. Biophys. Rep. 4, 89–97. doi: 10.1016/j.bbrep.2015.08.014 29124191 PMC5668897

[ref28] Eugénie N. Zivanovic Y. Lelandais G. Coste G. Bouthier de la Tour C. Bentchikou E.. (2021). Characterization of the radiation desiccation response regulon of the radioresistant bacterium *Deinococcus radiodurans* by integrative genomic analyses. Cells 10:2536. doi: 10.3390/cells10102536, PMID: 34685516 PMC8533742

[ref29] Farabaugh P. J. (1996). Programmed translational frameshifting. Microbiol. Rev. 60, 103–134. doi: 10.1128/mr.60.1.103-134.1996, PMID: 8852897 PMC239420

[ref30] Farci D. Bowler M. W. Esposito F. McSweeney S. Tramontano E. Piano D. (2015). Purification and characterization of DR_2577 (SlpA) a major S-layer protein from *Deinococcus radiodurans*. Front. Microbiol. 6:414. doi: 10.3389/fmicb.2015.00414, PMID: 26074883 PMC4419837

[ref31] Farci D. Esposito F. El Alaoui S. Piano D. (2017). S-layer proteins as a source of carotenoids: isolation of the carotenoid cofactor deinoxanthin from its S-layer protein DR_2577. Food Res. Int. 99, 868–876. doi: 10.1016/j.foodres.2016.10.003, PMID: 28847424

[ref32] Gaballa A. Newton G. L. Antelmann H. Parsonage D. Upton H. Rawat M.. (2010). Biosynthesis and functions of bacillithiol, a major low-molecular-weight thiol in Bacilli. Proc. Natl. Acad. Sci. U. S. A. 107, 6482–6486. doi: 10.1073/pnas.1000928107, PMID: 20308541 PMC2851989

[ref33] Galperin M. Y. Nikolskaya A. N. Koonin E. V. (2001). Novel domains of the prokaryotic two-component signal transduction systems. FEMS Microbiol. Lett. 203, 11–21. doi: 10.1111/j.1574-6968.2001.tb10814.x, PMID: 11557134

[ref34] Ghosal D. Omelchenko M. V. Gaidamakova E. K. Matrosova V. Y. Vasilenko A. Venkateswaran A.. (2005). How radiation kills cells: survival of *Deinococcus radiodurans* and *Shewanella oneidensis* under oxidative stress. FEMS Microbiol. Rev. 29, 361–375. doi: 10.1016/j.femsre.2004.12.007, PMID: 15808748

[ref35] Han J. M. Song H. Y. Jung J. H. Lim S. Seo H. S. Kim W. S.. (2023). *Deinococcus radiodurans*-derived membrane vesicles protect HaCaT cells against H_2_O_2_-induced oxidative stress via modulation of MAPK and Nrf2/ARE pathways. Biol. Proced. 25:17. doi: 10.1186/s12575-023-00211-4, PMID: 37328878 PMC10273539

[ref36] Hejna J. A. Moses R. E. (2009). “DNA replication” in Encyclopedia of microbiology, vol. 5. 3rd Edn. Ed. M. Schaechter (New York, USA: Academic Press), 113–122.

[ref37] Ho J. Adeolu M. Khadka B. Gupta R. S. (2016). Identification of distinctive molecular traits that are characteristic of the phylum “*Deinococcus-Thermus*” and distinguish its main constituent groups. Syst. Appl. Microbiol. 39, 453–463. doi: 10.1016/j.syapm.2016.07.003, PMID: 27506333

[ref38] Hua X. Hua Y. (2016). Improved complete genome sequence of the extremely radioresistant bacterium *Deinococcus radiodurans* R1 obtained using PacBio single-molecule sequencing. Genome Announc. 4:e00886-16. doi: 10.1128/genomeA.00886-16, PMID: 27587813 PMC5009970

[ref39] Hwang W. C. Bakolitsa C. Punta M. Coggill P. C. Bateman A. Axelrod H. L.. (2013). LUD, a new protein domain associated with lactate utilization. BMC Bioinformatics 14:341. doi: 10.1186/1471-2105-14-341 24274019 PMC3924224

[ref40] Im S. Song D. Joe M. Kim D. Park D. H. Lim S. (2013). Comparative survival analysis of 12 histidine kinase mutants of *Deinococcus radiodurans* after exposure to DNA-damaging agents. Bioprocess Biosyst. Eng. 36, 781–789. doi: 10.1007/s00449-013-0904-8, PMID: 23355081

[ref41] Iranzo J. Wolf Y. I. Koonin E. V. Sela I. (2019). Gene gain and loss push prokaryotes beyond the homologous recombination barrier and accelerate genome sequence divergence. Nat. Commun. 10:5376. doi: 10.1038/s41467-019-13429-2, PMID: 31772262 PMC6879757

[ref42] Islam S. M. Hua Y. Ohba H. Satoh K. Kikuchi M. Yanagisawa T.. (2003). Characterization and distribution of IS*8301* in the radioresistant bacterium *Deinococcus radiodurans*. Genes Genet. Syst. 78, 319–327. doi: 10.1266/ggs.78.319, PMID: 14676423

[ref43] Ithurbide S. Coste G. Lisboa J. Eugénie N. Bentchikou E. Bouthier de la Tour C.. (2020). Natural transformation in *Deinococcus radiodurans*: a genetic analysis reveals the major roles of DprA, DdrB, RecA, RecF, and RecO proteins. Front. Microbiol. 11:1253. doi: 10.3389/fmicb.2020.01253 32625182 PMC7314969

[ref44] Jack D. L. Yang N. M. Saier M. H. Jr. (2001). The drug/metabolite transporter superfamily. Eur. J. Biochem. 268, 3620–3639. doi: 10.1046/j.1432-1327.2001.02265.x 11432728

[ref45] Jang S. (2023). AcrAB−TolC, a major efflux pump in gram-negative bacteria: toward understanding its operation mechanism. BMB Rep. 56, 326–334. doi: 10.5483/BMBRep.2023-0070, PMID: 37254571 PMC10315565

[ref46] Jaspard E. (2006). A computational analysis of the three isoforms of glutamate dehydrogenase reveals structural features of the isoform EC 1.4.1.4 supporting a key role in ammonium assimilation by plants. Biol. Direct 1:38. doi: 10.1186/1745-6150-1-38, PMID: 17173671 PMC1716157

[ref47] Jeong S. Jung J. H. Kim M. K. de Groot A. Blanchard L. Ryu S.. (2021). Atypical bacilliredoxin AbxC plays a role in responding to oxidative stress in radiation-resistant bacterium *Deinococcus radiodurans*. Antioxidants 10:1148. doi: 10.3390/antiox10071148 34356381 PMC8301015

[ref48] Jeong S. W. Kim J. H. Kim J. W. Kim C. Y. Kim S. Y. Choi Y. J. (2020). Metabolic engineering of extremophilic bacterium *Deinococcus radiodurans* for the production of the novel carotenoid deinoxanthin. Microorganisms 9:44. doi: 10.3390/microorganisms9010044, PMID: 33375757 PMC7823818

[ref49] Karvelis T. Druteika G. Bigelyte G. Budre K. Zedaveinyte R. Silanskas A.. (2021). TnpB is a programmable RNA-guided DNA endonuclease. Nature 599, 692–696. doi: 10.1038/s41586-021-04058-1, PMID: 34619744 PMC8612924

[ref50] Keshav G. C. Gyawali P. Balci H. Abeysirigunawardena S. (2020). Ribosomal RNA methyltransferase RsmC moonlights as an RNA chaperone. Chembiochem 21, 1885–1892. doi: 10.1002/cbic.201900708, PMID: 31972066

[ref51] Koren S. Walenz B. P. Berlin K. Miller J. R. Bergman N. H. Phillippy A. M. (2017). Canu: scalable and accurate long-read assembly via adaptive k-mer weighting and repeat separation. Genome Res. 27, 722–736. doi: 10.1101/gr.215087.116 28298431 PMC5411767

[ref52] Leblanc C. Vilter H. Fournier J. B. Delage L. Potin P. Rebuffet E.. (2015). Vanadium haloperoxidases: from the discovery 30 years ago to X-ray crystallographic and V K-edge absorption spectroscopic studies. Coord. Chem. Rev. 301-302, 134–146. doi: 10.1016/j.ccr.2015.02.013

[ref53] Li H. Durbin R. (2010). Fast and accurate long-read alignment with burrows-wheeler transform. Bioinformatics 26, 589–595. doi: 10.1093/bioinformatics/btp698, PMID: 20080505 PMC2828108

[ref54] Lim S. Jung J. H. Blanchard L. de Groot A. (2019). Conservation and diversity of radiation and oxidative stress resistance mechanisms in *Deinococcus* species. FEMS Microbiol. Rev. 43, 19–52. doi: 10.1093/femsre/fuy037, PMID: 30339218 PMC6300522

[ref55] Liu Y. Gao Z. Q. She Z. Qu K. Wang W. J. Shtykova E. V.. (2012). The structural basis of the response regulator DrRRA from *Deinococcus radiodurans*. Biochem. Biophys. Res. Commun. 417, 1206–1212. doi: 10.1016/j.bbrc.2011.12.110 22227191

[ref56] Liu C. Wang L. Li T. Lin L. Dai S. Tian B.. (2014). A PerR-like protein involved in response to oxidative stress in the extreme bacterium *Deinococcus radiodurans*. Biochem. Biophys. Res. Commun. 450, 575–580. doi: 10.1016/j.bbrc.2014.06.015, PMID: 24928392

[ref57] Long H. Kucukyildirim S. Sung W. Williams E. Lee H. Ackerman M.. (2015). Background mutational features of the radiation-resistant bacterium *Deinococcus radiodurans*. Mol. Biol. Evol. 32, 2383–2392. doi: 10.1093/molbev/msv119, PMID: 25976352 PMC5009958

[ref58] Makarova K. S. Aravind L. Wolf Y. I. Tatusov R. L. Minton K. W. Koonin E. V.. (2001). Genome of the extremely radiation-resistant bacterium *Deinococcus radiodurans* viewed from the perspective of comparative genomics. Microbiol. Mol. Biol. Rev. 65, 44–79. doi: 10.1128/MMBR.65.1.44-79.2001, PMID: 11238985 PMC99018

[ref59] Manobala T. Shukla S. K. Rao T. S. Kumar M. D. (2019). A new uranium bioremediation approach using radio-tolerant *Deinococcus radiodurans* biofilm. J. Biosci. 44:122. doi: 10.1007/s12038-019-9942-y 31719231

[ref60] Maurya G. K. Modi K. Banerjee M. Chaudhary R. Rajpurohit Y. S. Misra H. S. (2018). Phosphorylation of FtsZ and FtsA by a DNA damage-responsive Ser/Thr protein kinase affects their functional interactions in *Deinococcus radiodurans*. mSphere 3:e00325-18. doi: 10.1128/mSphere.00325-18, PMID: 30021877 PMC6052341

[ref61] Maurya G. K. Modi K. Misra H. S. (2016). Divisome and segrosome components of *Deinococcus radiodurans* interact through cell division regulatory proteins. Microbiology 162, 1321–1334. doi: 10.1099/mic.0.000330, PMID: 27368754

[ref62] McCown P. J. Ruszkowska A. Kunkler C. N. Breger K. Hulewicz J. P. Wang M. C.. (2020). Naturally occurring modified ribonucleosides. Wiley Interdiscip. Rev. RNA. 11:e1595. doi: 10.1002/wrna.1595, PMID: 32301288 PMC7694415

[ref63] Mennecier S. Coste G. Servant P. Bailone A. Sommer S. (2004). Mismatch repair ensures fidelity of replication and recombination in the radioresistant organism *Deinococcus radiodurans*. Mol. Gen. Genomics. 272, 460–469. doi: 10.1007/s00438-004-1077-6, PMID: 15503140

[ref64] Mennecier S. Servant P. Coste G. Bailone A. Sommer S. (2006). Mutagenesis via IS transposition in *Deinococcus radiodurans*. Mol. Microbiol. 59, 317–325. doi: 10.1111/j.1365-2958.2005.04936.x, PMID: 16359337

[ref65] Miñambres B. Olivera E. R. Jensen R. A. Luengo J. M. (2000). A new class of glutamate dehydrogenases (GDH). Biochemical and genetic characterization of the first member, the AMP-requiring NAD-specific GDH of *Streptomyces clavuligerus*. J. Biol. Chem. 275, 39529–39542. doi: 10.1074/jbc.M005136200, PMID: 10924516

[ref66] Mishra S. Misra H. S. Kota S. (2022). FtsK, a DNA motor protein, coordinates the genome segregation and early cell division processes in *Deinococcus radiodurans*. mBio 13:e0174222. doi: 10.1128/mbio.01742-22, PMID: 36300930 PMC9764985

[ref67] Misra H. S. Khairnar N. P. Kota S. Shrivastava S. Joshi V. P. Apte S. K. (2006). An exonuclease I-sensitive DNA repair pathway in *Deinococcus radiodurans*: a major determinant of radiation resistance. Mol. Microbiol. 59, 1308–1316. doi: 10.1111/j.1365-2958.2005.05005.x, PMID: 16430702

[ref68] Modi K. Misra H. S. (2014). Dr-FtsA, an actin homologue in *Deinococcus radiodurans* differentially affects Dr-FtsZ and Ec-FtsZ functions in vitro. PLoS One 9:e115918. doi: 10.1371/journal.pone.0115918, PMID: 25551229 PMC4281207

[ref69] Mosquera-Rendón J. Cárdenas-Brito S. Pineda J. D. Corredor M. Benítez-Páez A. (2014). Evolutionary and sequence-based relationships in bacterial AdoMet-dependent non-coding RNA methyltransferases. BMC. Res. Notes 7:440. doi: 10.1186/1756-0500-7-440, PMID: 25012753 PMC4119055

[ref70] Narumi I. Cherdchu K. Kitayama S. Watanabe H. (1997). The *Deinococcus radiodurans uvrA* gene: identification of mutation sites in two mitomycin-sensitive strains and the first discovery of insertion sequence element from deinobacteria. Gene 198, 115–126. doi: 10.1016/s0378-1119(97)00301-6 9370272

[ref71] Niiranen L. Lian K. Johnson K. A. Moe E. (2015). Crystal structure of the DNA polymerase III β subunit (β-clamp) from the extremophile *Deinococcus radiodurans*. BMC Struct. Biol. 15:5. doi: 10.1186/s12900-015-0032-6 25886944 PMC4350885

[ref72] Ohba H. Satoh K. Yanagisawa T. Narumi I. (2005). The radiation responsive promoter of the *Deinococcus radiodurans pprA* gene. Gene 363, 133–141. doi: 10.1016/j.gene.2005.07.035, PMID: 16203111

[ref73] Owttrim G. W. (2013). RNA helicases: diverse roles in prokaryotic response to abiotic stress. RNA Biol. 10, 96–110. doi: 10.4161/rna.22638 23093803 PMC3590241

[ref74] Park H. R. Lee J. H. Ji H. J. Lim S. Ahn K. B. Seo H. S. (2022). Radioprotection of deinococcal exopolysaccharide BRD125 by regenerating hematopoietic stem cells. Front. Oncol. 12:898185. doi: 10.3389/fonc.2022.898185 36226052 PMC9549790

[ref75] Park S. H. Sohn Y. J. Park S. J. Choi J. I. (2020). Effect of DR1558, a *Deinococcus radiodurans* response regulator, on the production of GABA in the recombinant *Escherichia coli* under low pH conditions. Microb. Cell Factories 19:64. doi: 10.1186/s12934-020-01322-3, PMID: 32156293 PMC7063819

[ref76] Parte A. C. Carbasse J. S. Meier-Kolthoff J. P. Reimer L. C. Göker M. (2020). List of prokaryotic names with standing in nomenclature (LPSN) moves to the DSMZ. Int. J. Syst. Evol. Microbiol. 70, 5607–5612. doi: 10.1099/ijsem.0.004332, PMID: 32701423 PMC7723251

[ref77] Pasternak C. Ton-Hoang B. Coste G. Bailone A. Chandler M. Sommer S. (2010). Irradiation-induced *Deinococcus radiodurans* genome fragmentation triggers transposition of a single resident insertion sequence. PLoS Genet. 6:e1000799. doi: 10.1371/journal.pgen.1000799, PMID: 20090938 PMC2806898

[ref78] Pei J. Grishin N. V. (2001). GGDEF domain is homologous to adenylyl cyclase. Proteins 42, 210–216. doi: 10.1002/1097-0134(20010201)42:2<210::aid-prot80>3.0.co;2-8, PMID: 11119645

[ref79] Pei A. Nossa C. W. Chokshi P. Blaser M. J. Yang L. Rosmarin D. M.. (2009). Diversity of 23S rRNA genes within individual prokaryotic genomes. PLoS One 4:e5437. doi: 10.1371/journal.pone.0005437, PMID: 19415112 PMC2672173

[ref80] Pengpeng W. Tan Z. (2013). Ammonia assimilation in rumen bacteria: a review. Anim. Biotechnol. 24, 107–128. doi: 10.1080/10495398.2012.756402 23534958

[ref81] Periwal V. Scaria V. (2015). Insights into structural variations and genome rearrangements in prokaryotic genomes. Bioinformatics 31, 1–9. doi: 10.1093/bioinformatics/btu600, PMID: 25189783

[ref82] Petchiappan A. Naik S. Y. Chatterji D. (2020). Tracking the homeostasis of second messenger cyclic-di-GMP in bacteria. Biophys. Rev. 12, 719–730. doi: 10.1007/s12551-020-00636-1, PMID: 32060735 PMC7311556

[ref83] Pichoff S. Du S. Lutkenhaus J. (2019). Roles of FtsEX in cell division. Res. Microbiol. 170, 374–380. doi: 10.1016/j.resmic.2019.07.003 31376483 PMC6899183

[ref84] Razew A. Schwarz J. N. Mitkowski P. Sabala I. Kaus-Drobek M. (2022). One fold, many functions-M23 family of peptidoglycan hydrolases. Front. Microbiol. 13:1036964. doi: 10.3389/fmicb.2022.1036964, PMID: 36386627 PMC9662197

[ref85] Reitzer L. (2014). “Amino acid synthesis” in Reference module in biomedical sciences. Ed. M. J. Caplan (Amsterdam: Elsevier).

[ref86] Repar J. Zahradka D. Sović I. Zahradka K. (2021). Characterization of gross genome rearrangements in *Deinococcus radiodurans* recA mutants. Sci. Rep. 11:10939. doi: 10.1038/s41598-021-89173-9, PMID: 34035321 PMC8149714

[ref87] Richards J. Belasco J. G. (2011). Ribonuclease J: how to lead a double life. Structure 19, 1201–1203. doi: 10.1016/j.str.2011.08.004 21893280 PMC3176637

[ref88] Richter M. Rosselló-Móra R. Oliver Glöckner F. Peplies J. (2016). JSpeciesWS: a web server for prokaryotic species circumscription based on pairwise genome comparison. Bioinformatics 32, 929–931. doi: 10.1093/bioinformatics/btv681 26576653 PMC5939971

[ref89] Santos-Beneit F. (2015). The pho regulon: a huge regulatory network in bacteria. Front. Microbiol. 6:402. doi: 10.3389/fmicb.2015.00402, PMID: 25983732 PMC4415409

[ref90] Schindler B. D. Kaatz G. W. (2016). Multidrug efflux pumps of gram-positive bacteria. Drug Resist. Updat. 27, 1–13. doi: 10.1016/j.drup.2016.04.003 27449594

[ref91] Schmier B. J. Seetharaman J. Deutscher M. P. Hunt J. F. Malhotra A. (2012). The structure and enzymatic properties of a novel RNase II family enzyme from *Deinococcus radiodurans*. J. Mol. Biol. 415, 547–559. doi: 10.1016/j.jmb.2011.11.031 22133431 PMC3269974

[ref92] Seemann T. (2014). Prokka: rapid prokaryotic genome annotation. Bioinformatics 30, 2068–2069. doi: 10.1093/bioinformatics/btu153 24642063

[ref93] Sharma A. Gaidamakova E. K. Grichenko O. Matrosova V. Y. Hoeke V. Klimenkova P.. (2017). Across the tree of life, radiation resistance is governed by antioxidant Mn^2+^, gauged by paramagnetic resonance. Proc. Natl. Acad. Sci. U. S. A. 114, E9253–E9260. doi: 10.1073/pnas.1713608114 29042516 PMC5676931

[ref94] Slade D. Radman M. (2011). Oxidative stress resistance in *Deinococcus radiodurans*. Microbiol. Mol. Biol. Rev. 75, 133–191. doi: 10.1128/MMBR.00015-10, PMID: 21372322 PMC3063356

[ref95] Southworth M. W. Perler F. B. (2002). Protein splicing of the *Deinococcus radiodurans* strain R1 Snf2 intein. J. Bacteriol. 184, 6387–6388. doi: 10.1128/JB.184.22.6387-6388.2002, PMID: 12399510 PMC151952

[ref96] Steczkiewicz K. Muszewska A. Knizewski L. Rychlewski L. Ginalski K. (2012). Sequence, structure and functional diversity of PD-(D/E)XK phosphodiesterase superfamily. Nucleic Acids Res. 40, 7016–7045. doi: 10.1093/nar/gks382, PMID: 22638584 PMC3424549

[ref97] Takeda M. Anamizu S. Motomatsu S. Chen X. Thapa C. R. (2014). Identification and characterization of a mycobacterial NAD^+^-dependent alcohol dehydrogenase with superior reduction of diacetyl to (*S*)-acetoin. Biosci. Biotechnol. Biochem. 78, 1879–1886. doi: 10.1080/09168451.2014.943649 25082080

[ref98] Tarasov A. Vilella A. J. Cuppen E. Nijman I. J. Prins P. (2015). Sambamba: fast processing of NGS alignment formats. Bioinformatics 31, 2032–2034. doi: 10.1093/bioinformatics/btv098, PMID: 25697820 PMC4765878

[ref99] Tatusova T. DiCuccio M. Badretdin A. Chetvernin V. Nawrocki E. P. Zaslavsky L.. (2016). NCBI prokaryotic genome annotation pipeline. Nucleic Acids Res. 44, 6614–6624. doi: 10.1093/nar/gkw569 27342282 PMC5001611

[ref100] Timmins J. Moe E. (2016). A decade of biochemical and structural studies of the DNA repair machinery of *Deinococcus radiodurans*: major findings, functional and mechanistic insight and challenges. Comput. Struct. Biotechnol. J. 14, 168–176. doi: 10.1016/j.csbj.2016.04.001, PMID: 27924191 PMC5128194

[ref101] Vale F. F. Lehours P. Yamaoka Y. (2022). Editorial: the role of mobile genetic elements in bacterial evolution and their adaptability. Front. Microbiol. 13:849667. doi: 10.3389/fmicb.2022.849667, PMID: 35265063 PMC8899501

[ref102] Venkateswaran A. McFarlan S. C. Ghosal D. Minton K. W. Vasilenko A. Makarova K.. (2000). Physiologic determinants of radiation resistance in *Deinococcus radiodurans*. Appl. Environ. Microbiol. 66, 2620–2626. doi: 10.1128/AEM.66.6.2620-2626.2000, PMID: 10831446 PMC110589

[ref103] Vigouroux A. Cordier B. Aristov A. Alvarez L. Özbaykal G. Chaze T.. (2020). Class-a penicillin binding proteins do not contribute to cell shape but repair cell-wall defects. Elife 9:e51998. doi: 10.7554/eLife.51998, PMID: 31904338 PMC7002073

[ref104] von Kügelgen A. van Dorst S. Alva V. Bharat T. A. M. (2022). A multidomain connector links the outer membrane and cell wall in phylogenetically deep-branching bacteria. Proc. Natl. Acad. Sci. U. S. A. 119:e2203156119. doi: 10.1073/pnas.2203156119, PMID: 35943982 PMC9388160

[ref105] Walker B. J. Abeel T. Shea T. Priest M. Abouelliel A. Sakthikumar S.. (2014). Pilon: an integrated tool for comprehensive microbial variant detection and genome assembly improvement. PLoS One 9:e112963. doi: 10.1371/journal.pone.0112963, PMID: 25409509 PMC4237348

[ref106] Wang L. Tan Y. S. Chen K. Ntakirutimana S. Liu Z. H. Li B. Z.. (2024). Global regulator IrrE on stress tolerance: a review. Crit. Rev. Biotechnol., 1–21. doi: 10.1080/07388551.2023.2299766, PMID: 38246753

[ref107] Wang L. Xu G. Chen H. Zhao Y. Xu N. Tian B.. (2008). DrRRA: a novel response regulator essential for the extreme radioresistance of *Deinococcus radiodurans*. Mol. Microbiol. 67, 1211–1222. doi: 10.1111/j.1365-2958.2008.06113.x, PMID: 18208531

[ref108] White O. Eisen J. A. Heidelberg J. F. Hickey E. K. Peterson J. D. Dodson R. J.. (1999). Genome sequence of the radioresistant bacterium *Deinococcus radiodurans* R1. Science 286, 1571–1577. doi: 10.1126/science.286.5444.1571, PMID: 10567266 PMC4147723

[ref109] Willems P. Fijalkowski I. Van Damme P. (2020). Lost and found: re-searching and re-scoring proteomics data aids genome annotation and improves proteome coverage. mSystems 5:e00833-20. doi: 10.1128/mSystems.00833-20, PMID: 33109751 PMC7593589

[ref110] Xiao Z. Xu P. (2007). Acetoin metabolism in bacteria. Crit. Rev. Microbiol. 33, 127–140. doi: 10.1080/10408410701364604 17558661

[ref111] Yang S. Xu H. Wang J. Liu C. Lu H. Liu M.. (2016). Cyclic AMP receptor protein acts as a transcription regulator in response to stresses in *Deinococcus radiodurans*. PLoS One 11:e0155010. doi: 10.1371/journal.pone.0155010, PMID: 27182600 PMC4868304

[ref112] Ying X. Ma K. (2011). Characterization of a zinc-containing alcohol dehydrogenase with stereoselectivity from the hyperthermophilic archaeon *Thermococcus guaymasensis*. J. Bacteriol. 193, 3009–3019. doi: 10.1128/JB.01433-10, PMID: 21515780 PMC3133181

[ref113] Yu J. Li T. Dai S. Weng Y. Li J. Li Q.. (2017). A *tamB* homolog is involved in maintenance of cell envelope integrity and stress resistance of *Deinococcus radiodurans*. Sci. Rep. 7:45929. doi: 10.1038/srep45929, PMID: 28383523 PMC5382914

[ref114] Zahradka K. Slade D. Bailone A. Sommer S. Averbeck D. Petranovic M.. (2006). Reassembly of shattered chromosomes in *Deinococcus radiodurans*. Nature 443, 569–573. doi: 10.1038/nature05160, PMID: 17006450

