# Supplementary material for: Comparative genomics of Deinococcus radiodurans: unveiling genetic discrepancies between ATCC 13939K and BAA-816 strains
Source: Front Microbiol. 2024 Jun 19;15:1410024. doi: 10.3389/fmicb.2024.1410024 (PMC11219805; doi:10.3389/fmicb.2024.1410024)
Supplement: Supplementary file 6 [file Data_Sheet_1.pdf]

```
DR_2566            1              M  P  R  L  T  V  R  P  A  G  R  R  A  R  A  G  F  F  L  T  S  I  S  S  G  R  R  K  T  G  A  D  F  A  A  R
BAA-816            1  atgcccctgggATGCCTCGCCTCACTGTTCGGCCTGCGGGACGCCGCGCCCGCGCCGGCTTCTTCCTCACCTCCATCTCCTCTGGCCGGCGAAAAACGGGTGCCGACTTCGCTGCCCGTC  120
                      ||||||||||||||||||||||||||||||||||||||||||||||||||||||||||||||||||||||||||||||||||||||||||||||||||||||||||||||||||||||||
ATCC 13939K        1  ATGCCCCTGGGATGCCTCGCCTCACTGTTCGGCCTGCGGGACGCCGCGCCCGCGCCGGCTTCTTCCTCACCTCCATCTCCTCTGGCCGGCGAAAAACGGGTGCCGACTTCGCTGCCCGTC  120
KDR_2566           1   M  P  L  G  C  L  A  S  L  F  G  L  R  D  A  A  P  A  P  A  S  S  S  P  P  S  P  L  A  G  E  K  R  V  P  T  S  L  P  V

DR_2566           37  L  G  A  A  L  L  L  F  R  R  R  E  R  F  F  S  R  S  G  R  R   L  R  G  T  S  Y  R  A  F  P  N  V  R  L  N  D  L  F  T
BAA-816          121  TCGGCGCGGCGCTACTTCTTTTCCGCCGACGAGAACGCtttttttCGCGCTCTGGAAGACG-GCTGCGCGGCACGAGCTACCGGGCCTTTCCCAATGTGCGGCTCAACGACCTCTTTACC  239
                      ||||||||||||||||||||||||||||||||||||||||||||||||||||||||||||| ||||||||||||||||||||||||||||||||||||||||||||||||||||||||||
ATCC 13939K      121  TCGGCGCGGCGCTACTTCTTTTCCGCCGACGAGAACGCTTTTTTTCGCGCTCTGGAAGACGCGCTGCGCGGCACGAGCTACCGGGCCTTTCCCAATGTGCGGCTCAACGACCTCTTTACC  240
KDR_2566          41   S  A  R  R  Y  F  F  S  A  D  E  N  A  F  F  R  A  L  E  D  A  L  R  G  T  S  Y  R  A  F  P  N  V  R  L  N  D  L  F  T

DR_2566          137   S  G  G  L  T  L  L  R  L  P  S  R  R  Y  S  V  A  E  L  R  N  E  L  R  R  A  G  L  K  S  G  N  *
BAA-816          420  TCCGGGGGCCTCACGCTGCTGCGGCTGCCTTCGCGCCGCTACTCGGTGGCGGAACTCAGAAACGAACTCAGACGCGCTGGCCTCAAGTCGGGCAACTGA  518
                      |||||||||||||||||||||||||||||||||||||||||||||||||||||||||||||||||||||||||||||||||||||||||||||||||||
ATCC 13939K      420  TCCGGGGGCCTCACGCTGCTGCGGCTGCCTTCGCGCCGCTACTCGGTGGCGGAACTCAGAAACGAACTCAGACGCGCTGGCCTCAAGTCGGGCAACTGA  519
KDR_2566         141   S  G  G  L  T  L  L  R  L  P  S  R  R  Y  S  V  A  E  L  R  N  E  L  R  R  A  G  L  K  S  G  N  *
```

Figure S1. Comparison of nucleotide and amino acid sequences for a putative VSR-like nuclease between the BAA-816 and ATCC 13939K strains. Sequence numbering of nucleotides is based on the start codon of the *KDR_2566* gene. The corresponding amino acid sequences are displayed above (for BAA-816) and below (for ATCC 13939K) the nucleotide sequences, starting from the N-terminus of each protein and marked on the left. Non-coding regions identified within the BAA-816 genome are depicted in lowercase letters. Any nucleotide addition found in the sequences is highlighted in bold. A stop codon is denoted by an asterisk.

```
DR_2410          1   M  S  A  I  Y  Q  R  A  R  P  I  R  W  E  D  V  V  G  Q  E  H  V  K  D  V  L  R  T  A  L  E  Q  G  R  I  G  H  A  Y  L
BAA-816          1  ATGAGCGCCATCTATCAGCGGGCGCGGCCTATCCGCTGGGAAGATGTCGTGGGCCAGGAACACGTCAAGGACGTGTTGCGCACCGCGCTGGAGCAGGGGCGCATCGGGCACGCCTACCTG   120
                    ||||||||||||||||||||||||||||||||||||||||||||||||||||||||||||||||||||||||||||||||||||||||||||||||||||||||||||||||||||||||||
ATCC 13939K      1  ATGAGCGCCATCTATCAGCGGGCGCGGCCTATCCGCTGGGAAGATGTCGTGGGCCAGGAACACGTCAAGGACGTGTTGCGCACCGCGCTGGAGCAGGGGCGCATCGGGCACGCCTACCTG   120
KDR_2410m        1   M  S  A  I  Y  Q  R  A  R  P  I  R  W  E  D  V  V  G  Q  E  H  V  K  D  V  L  R  T  A  L  E  Q  G  R  I  G  H  A  Y  L

DR_2410        581   Q  T  P  G  T  Q  T  A  S  A  P  P  P  S  L  P  P  R  P  P  A  R  P  S  G  T  R  P  C  A  C  G  P  A  T  *
BAA-816       1741  CAGACCCCCGGCACTCAGACGGCCAGCGCCCCACCGCCCAGCCTGCCGCCGCGCCCCCCGGCCCGGCCCAGTGGGACTCGCCCGTGCGCCTGCGGCCCGGCAACCTGAactccgcctgcc  1860
                    |||||||||||||||||||||||||||||||||||||||||||||||||||||||||||||||||||||||||||||||||||| |||||||||||||||||||||||||||||||||||
ATCC 13939K   1741  CAGACCCCCGGCACTCAGACGGCCAGCGCCCCACCGCCCAGCCTGCCGCCGCGCCCCCCGGCCCGGCCCAGTGGGACTCGCCCG-GCGCCTGCGGCCCGGCAACCTGAACTCCGCCTGCC  1859
KDR_2410m      581   Q  T  P  G  T  Q  T  A  S  A  P  P  P  S  L  P  P  R  P  P  A  R  P  S  G  T  R  P     A  P  A  A  R  Q  P  E  L  R  L  P

DR_2411          1                        M  A  P  A  P  L  P  G  N  E  A  A  P  W  Q  D  E  H  A  A  E  P  P  P  A  P  A  D  A  Q  G  G  D
BAA-816       1861  cgacccgcccagccccgacgacGTGGCCCCCGCGCCGCTGCCTGGGAACGAGGCCGCGCCCTGGCAGGACGAGCACGCCGCCGAGCCGCCCCCTGCCCCCGCCGACGCCCAGGGCGGTGA  1980
                    ||||||||||||||||||||||||||||||||||||||||||||||||||||||||||||||||||||||||||||||||||||||||||||||||||||||||||||||||||||||||
ATCC 13939K   1860  CGACCCGCCCAGCCCCGACGACGTGGCCCCCGCGCCGCTGCCTGGGAACGAGGCCGCGCCCTGGCAGGACGAGCACGCCGCCGAGCCGCCCCCTGCCCCCGCCGACGCCCAGGGCGGTGA  1979
KDR_2410m      621    D  P  P  S  P  D  D  V  A  P  A  P  L  P  G  N  E  A  A  P  W  Q  D  E  H  A  A  E  P  P  P  A  P  A  D  A  Q  G  G  D


DR_2411        134    G  K  N  R  R  V  Q  A  D  S  S  D  A  D  G  E  A  D  E  S  E  D  T  A  E  A  *
BAA-816       2281  CGGCAAGAACCGCCGGGTGCAGGCGGACAGCAGCGACGCCGATGGAGAAGCCGACGAGAGCGAAGACACCGCCGAAGCCTGA  2362
                    ||||||||||||||||||||||||||||||||||||||||||||||||||||||||||||||||||||||||||||||||||
ATCC 13939K   2280  CGGCAAGAACCGCCGGGTGCAGGCGGACAGCAGCGACGCCGATGGAGAAGCCGACGAGAGCGAAGACACCGCCGAAGCCTGA  2361
KDR_2410m      761    G  K  N  R  R  V  Q  A  D  S  S  D  A  D  G  E  A  D  E  S  E  D  T  A  E  A  *
```

Figure S2. Comparison of nucleotide and amino acid sequences for DnaX between the BAA-816 and ATCC 13939K strains. The numbering of nucleotide sequences originates from the initiation codons of the genes *DR_2410* and *KDR_2410m*. For common details, refer to the legend of Figure S1.

```
DR_1647          1  M  G  R  N  V  A  A  E  Y  N  K  G  T  L  G  E  F  L  R  L  S  P  R  D  V  Q  Q  A  R  A  E  R  R  P  E  L  D  R  V  A
BAA-816          1 ATGGGGCGGAACGTAGCGGCGGAATACAACAAAGGCACCTTGGGCGAGTTTCTCCGCCTCTCGCCGCGAGACGTGCAGCAGGCGCGGGCCGAGCGGCGCCCTGAGCTTGACCGGGTGGCC  120
                   ||||||||||||||||||||||||||||||||||||||||||||||||||||||||||||||||||||||||||||||||||||||||||||||||||||||||||||||||||||||||
ATCC 13939K      1 ATGGGGCGGAACGTAGCGGCGGAATACAACAAAGGCACCTTGGGCGAGTTTCTCCGCCTCTCGCCGCGAGACGTGCAGCAGGCGCGGGCCGAGCGGCGCCCTGAGCTTGACCGGGTGGCC  120
KDR_1647         1  M  G  R  N  V  A  A  E  Y  N  K  G  T  L  G  E  F  L  R  L  S  P  R  D  V  Q  Q  A  R  A  E  R  R  P  E  L  D  R  V  A

DR_1647        301  D  L  L  A  L  L  N  A  D  P  S  R  L  T  P  A  A  G   C  A  R  R  F  R  T  R  C  C  P  P  S  P  S  S  S  A  P  A  K  S
BAA-816        901 GACCTGCTTGCGCTGCTGAATGCCGACCCCTCCCGTCTGACCCCCGCCGCAGG-CTGCGCCCGGCGATTCAGGACACGCTGCTGCCCACCCTCGCCTTCGTCGTCGGCCCCGGCGAAATC  1019
                   ||||||||||||||||||||||||||||||||||||||||||||||||||||| ||||||||||||||||||||||||||||||||||||||||||||||||||||||||||||||||||
ATCC 13939K    901 GACCTGCTTGCGCTGCTGAATGCCGACCCCTCCCGTCTGACCCCCGCCGCAGGCCTGCGCCCGGCGATTCAGGACACGCTGCTGCCCACCCTCGCCTTCGTCGTCGGCCCCGGCGAAATC  1020
KDR_1647       301  D  L  L  A  L  L  N  A  D  P  S  R  L  T  P  A  A  G  L  R  P  A  I  Q  D  T  L  L  P  T  L  A  F  V  V  G  P  G  E  I

DR_1647        341   R  M  E  R  N  S  K  T  C  T  R  C  T  A  C  N  N  P  Y  C  G  R  A  *
BAA-816       1020 GCGTATGGAGCGCAACTCAAAAACGTGTACCCGCTGCACGGCTTGCAACAACCCCTACTGTGGCCGCGCCTGAgcgtgacctggctggaacccaatgtcgcccgcttgctgcgccgcctc  1139
                   ||||||||||||||||||||||||||||||||||||||||||||||||||||||||||||||||||||||||||||||||||||||||||||||||||||||||||||||||||||||||
ATCC 13939K   1021 GCGTATGGAGCGCAACTCAAAAACGTGTACCCGCTGCACGGCTTGCAACAACCCCTACTGTGGCCGCGCCTGAGCGTGACCTGGCTGGAACCCAATGTCGCCCGCTTGCTGCGCCGCCTC  1140
KDR_1647       341  A  Y  G  A  Q  L  K  N  V  Y  P  L  H  G  L  Q  Q  P  L  L  W  P  R  L  S  V  T  W  L  E  P  N  V  A  R  L  L  R  R  L


BAA-816       1500 accccgctccggcaactgctggcgctggacgcggggttcgtaggagaagtcgagattccgtaa  1562
                   |||||||||||||||||||||||||||||||||||||||||||||||||||||||||||||||
ATCC 13939K   1501 ACCCCGCTCCGGCAACTGCTGGCGCTGGACGCGGGGTTCGTAGGAGAAGTCGAGATTCCGTAA  1563
KDR_1647       501  T  P  L  R  Q  L  L  A  L  D  A  G  F  V  G  E  V  E  I  P  *
```

Figure S3. Comparison of nucleotide and amino acid sequences for BshC between the BAA-816 and ATCC 13939K strains. The numbering of nucleotide sequences originates from the initiation codons of the genes *DR_1647* and *KDR_1647*. For common details, refer to the legend of Figure S1.

```
DR_1015          1   M  S  R  A  S  L  S  H  W  Q  P  H  F  R  R  L  A  R  W  C  A  V  S  V  C  L  G  I  A  A  G  A  G  A  V  A  Q  S  S  A
BAA-816          1  ATGTCGCGTGCTTCCCTGTCCCACTGGCAGCCCCACTTCCGGCGGCTGGCCCGCTGGTGCGCCGTGAGTGTCTGCCTGGGAATCGCCGCTGGTGCGGGCGCGGTGGCTCAGAGCTCAGCA  120
                    ||||||||||||||||||||||||||||||||||||||||||||||||||||||||||||||||||||||||||||||||||||||||||||||||||||||||||||||||||||||||
ATCC 13939K      1  ATGTCGCGTGCTTCCCTGTCCCACTGGCAGCCCCACTTCCGGCGGCTGGCCCGCTGGTGCGCCGTGAGTGTCTGCCTGGGAATCGCCGCTGGTGCGGGCGCGGTGGCTCAGAGCTCAGCA  120
KDR_1014m        1   M  S  R  A  S  L  S  H  W  Q  P  H  F  R  R  L  A  R  W  C  A  V  S  V  C  L  G  I  A  A  G  A  G  A  V  A  Q  S  S  A

DR_1015        241   E  P  G  W  G  R  V  Q  P  I  G  L  A  P  D  A  L  P  Q   S  R  R  P  P  A  G  R  A  P  S  S  P  P  T  A  S  V  S  P  T
BAA-816        721  GAACCCGGCTGGGGACGGGTGCAGCCCATCGGCCTCGCCCCGGACGCACTGCCGCAG-TCACGCCGCCCCCCGGCTGGCAGAGCGCCGAGTTCACCGCCGACCGCGAGCGTTTCGCCGAC  839
                    ||||||||||||||||||||||||||||||||||||||||||||||||||||||||| ||||||||||||||||||||||||||||||||||||||||||||||||||||||||||||||
ATCC 13939K    721  GAACCCGGCTGGGGACGGGTGCAGCCCATCGGCCTCGCCCCGGACGCACTGCCGCAGGTCACGCCGCCCCCCGGCTGGCAGAGCGCCGAGTTCACCGCCGACCGCGAGCGTTTCGCCGAC  840
KDR1014m       241   E  P  G  W  G  R  V  Q  P  I  G  L  A  P  D  A  L  P  Q  V  T  P  P  P  G  W  Q  S  A  E  F  T  A  D  R  E  R  F  A  D

DR_1015        281    S  R  P  D  *
DR_1014          1                                                                         M  T  P  L  G  L  W  I  E  E  A  L  R  L  G  E  Q  A
BAA-816        840  CAGCAGACCCGACTGAccccggcggaccgcgccctggccgcccactgggctgccgggtcgggcagcGTGACCCCGCTGGGGCTGTGGATCGAAGAAGCGCTGCGGCTGGGCGAGCAGGCG  959
                    ||||||||||||||||||||||||||||||||||||||||||||||||||||||||||||||||||||||||||||||||||||||||||||||||||||||||||||||||||||||||
ATCC 13939K    841  CAGCAGACCCGACTGACCCCGGCGGACCGCGCCCTGGCCGCCCACTGGGCTGCCGGGTCGGGCAGCGTGACCCCGCTGGGGCTGTGGATCGAAGAAGCGCTGCGGCTGGGCGAGCAGGCG  960
KDR_1014m      281   Q  Q  T  R  L  T  P  A  D  R  A  L  A  A  H  W  A  A  G  S  G  S  V  T  P  L  G  L  W  I  E  E  A  L  R  L  G  E  Q  A


DR_1014        119   G  I  H  W  G  V  D  G  V  A  G  L  D  V  G  Q  R  V  A  R  A  L  L  E  K  R  P  *
BAA-816       1260  GGCATCCACTGGGGCGTGGACGGCGTGGCGGGCCTCGACGTAGGGCAACGGGTGGCGCGGGCGTTGCTGGAAAGCGGCCCTGA  1343
                    |||||||||||||||||||||||||||||||||||||||||||||||||||||||||||||||||||||||||||||||||||
ATCC 13939K   1261  GGCATCCACTGGGGCGTGGACGGCGTGGCGGGCCTCGACGTAGGGCAACGGGTGGCGCGGGCGTTGCTGGAAAGCGGCCCTGA  1344
KDR_1014m      421   G  I  H  W  G  V  D  G  V  A  G  L  D  V  G  Q  R  V  A  R  A  L  L  E  K  R  P  *
```

Figure S4. Comparison of nucleotide and amino acid sequences for V-HPO between the BAA-816 and ATCC 13939K strains. The numbering of nucleotide sequences originates from the initiation codons of the genes *DR_1015* and *KDR_1014m*. For common details, refer to the legend of Figure S1.

```
BAA-816           1  ttgaaacgtaccctgctccccgccctgctgctcgcctcgttcgctgccgcgcaaaccgccgcgcccgcagctccctcgcctacgccggcgcccgccagcgcgacccgactgctgccgcct  120
                     ||||||||||||||||||||||||||||||||||||||||||||||||||||||||||||||||||||||||||||||||||||||||||||||||||||||||||||||||||||||||
ATCC 13939K       1  TTGAAACGTACCCTGCTCCCCGCCCTGCTGCTCGCCTCGTTCGCTGCCGCGCAAACCGCCGCGCCCGCAGCTCCCTCGCCTACGCCGGCGCCCGCCAGCGCGACCCGACTGCTGCCGCCT  120
KDR_2567          1   M  K  R  T  L  L  P  A  L  L  L  A  S  F  A  A  A  Q  T  A  A  P  A  A  P  S  P  T  P  A  P  A  S  A  T  R  L  L  P  P

DR_2567           1                                        M  V  G  E  E  G  G  T  Y  T  V  Q  V  S  D  G  L  T  L  N  A  P  K  S  S  L  T  L
BAA-816         781  ttcgtggtctagccccgccccggcgcccggaccatcGTGGTGGGCGAGGAAGGGGGCACCTACACCGTGCAGGTGTCGGATGGCCTGACGCTCAACGCGCCGAAGAGCTCCCTGACGCTG  900
                     ||||||||||| ||||||||||||||||||||||||||||||||||||||||||||||||||||||||||||||||||||||||||||||||||||||||||||||||||||||||||||||
ATCC 13939K     781  TTCGTGGTCTACCCCCGCCCCGGCGCCCGGACCATCGTGGTGGGCGAGGAAGGGGGCACCTACACCGTGCAGGTGTCGGATGGCCTGACGCTCAACGCGCCGAAGAGCTCCCTGACGCTG  900
KDR_2567        291   F  V  V  Y  P  R  P  G  A  R  T  I  V  V  G  E  E  G  G  T  Y  T  V  Q  V  S  D  G  L  T  L  N  A  P  K  S  S  L  T  L

DR_2567         329   A  L  G  L  E  R  F  Y  R  D  A  A  L  K  Q  R  *
BAA-816        1801  GCGCTGGGGCTGGAGCGCTTTTACCGGGACGCGGCGCTGAAACAGCGGTAA  1851
                     |||||||||||||||||||||||||||||||||||||||||||||||||||
ATCC 13939K    1801  GCGCTGGGGCTGGAGCGCTTTTACCGGGACGCGGCGCTGAAACAGCGGTAA  1851
KDR_2567        601   A  L  G  L  E  R  F  Y  R  D  A  A  L  K  Q  R  *
```

Figure S5. Comparison of nucleotide and amino acid sequences for a putative amidase between the BAA-816 and ATCC 13939K strains. Sequence numbering of nucleotides is based on the start codon of the *KDR_2567* gene. A nucleotide variation between sequences is highlighted in bold. For common details, refer to the legend of Figure S1.

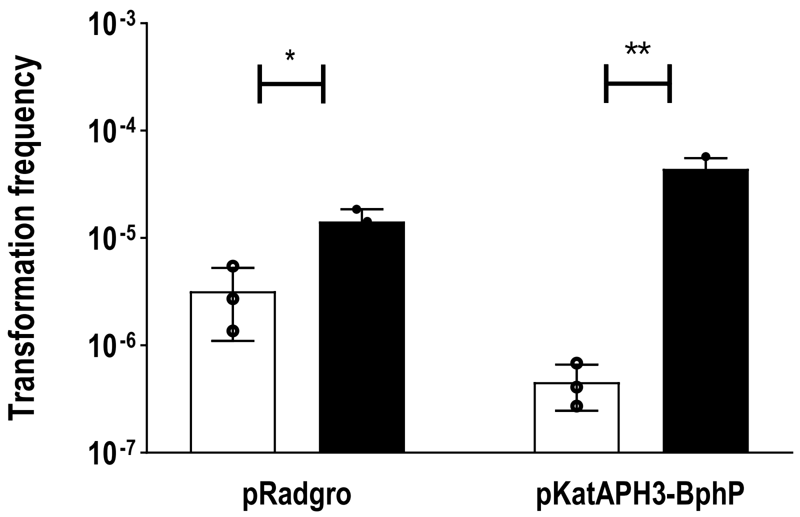

Figure S6. Comparison of the frequency of plasmid transformation between the ATCC 13939K (white bars) and BAA-816 strains (black bars). Cells were introduced to 250 ng of plasmid DNA. The rate of transformation was determined by the ratio of transformants to the total number of plated cells. The bar graph represents the mean ± SD (n=3). Statistical significance was assessed using an unpaired t test. *p < 0.05; ***p* < 0.01.

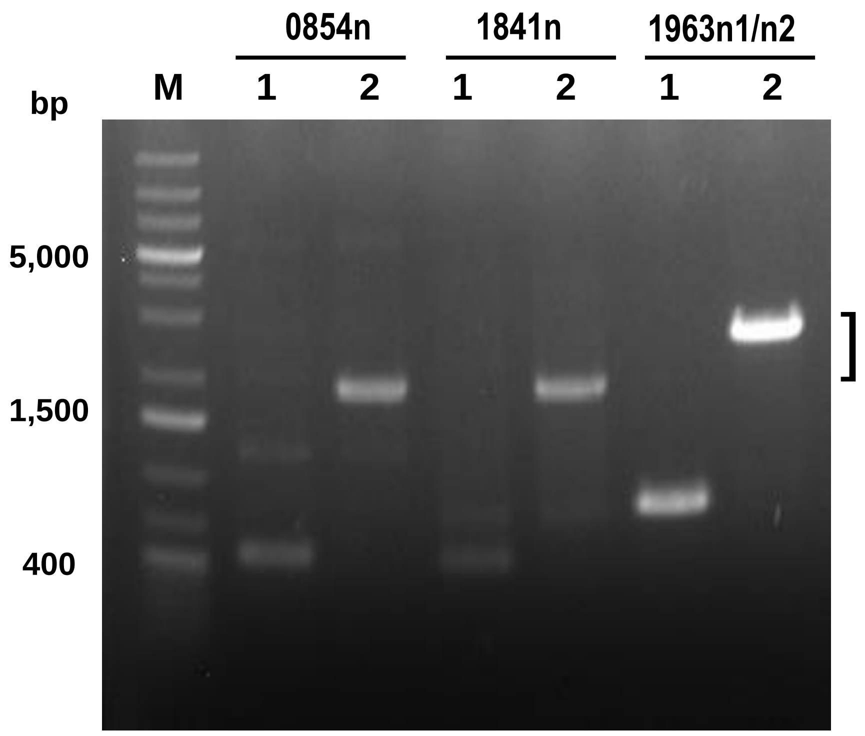

Figure S7. Detection of IS transposition in the ATCC 13939K strain. Genomic DNA samples were extracted from both BAA-816 (lane 1) and ATCC 13939K (lane 2) strains. PCR analyses were performed to ascertain the presence of IS*2621* (*KDR_0854n* and *KDR_1841n*) and IS*Dra2* (*KDR_1963n1* and *KDR_1963n2*). Notable insertions within the ATCC 13939K genome are marked with brackets, displaying fragment sizes of 1,929 bp (lane 2), 1,852 bp (lane 4), and 2,503 bp (lane 6). The first lane (M) indicates DNA size marker.

```
DR_A0270       1  MNSQDRDETNGDRQRLVRVALGQEDGDLLVRGAQVVQPVTREVYAADVLVAGGRVAAVGPDLPARARRTVQARGAYLAPGFIDGHIHIESSLLTPASFAA  100
                  ||||||||||||||||||||||||||||||||||||||||||||||||||||||||||||||||||||||||||||||||||||||||||||||||||||
KDR_A0270      1  MNSQDRDETNGDRQRLVRVALGQEDGDLLVRGAQVVQPVTREVYAADVLVAGGRVAAVGPDLPARARRTVQARGAYLAPGFIDGHIHIESSLLTPASFAA  100

DR_A0270     101  AVLPHGTTAVVAEPHEIVNVLGPAGLNWMLGAGRTSGLRVYASAPSCVPASEFEQGGARVDAAQVAEMLARPGVLGLAEMMNYPGVLGGDAGVWDILNAG  200
                  ||||||||||||||||||||||||||||||||||||||||||||||||||||||||||||||||||||||||||||||||||||||||||||||||||||
KDR_A0270    101  AVLPHGTTAVVAEPHEIVNVLGPAGLNWMLGAGRTSGLRVYASAPSCVPASEFEQGGARVDAAQVAEMLARPGVLGLAEMMNYPGVLGGDAGVWDILNAG  200
DR_RS17005     1                                                     MTLGLYVPRDSPLHRAAPGVKLLGLLLCGVLVFMLHDWRPLLALLGLTL   49

DR_A0270     201  RRSGKRLDGHAAGLGGRELLAYAAAGLHSDHEATTPEEARERLRAGLWLMVREGSAARNLAALLPVLRDLPRRALLVSDDVSVDELLELGHLDRLLRTCV  300
                  ||||||||||||||||||||||||||||||||||||||||||||||||||||||||||||||||||||||||||||||||||||||||||||||||||||
KDR_A0270    201  RRSGKRLDGHAAGLGGRELLAYAAAGLHSDHEATTPEEARERLRAGLWLMVREGSAARNLAALLPVLRDLPRRALLVSDDVSVDELLELGHLDRLLRTCV  300
DR_RS17005    50  ALYAVARLGWRTTWAQLRPALGLLLFFLVVXGLLTNWETAVVTALRFGVMILLASLVTLTTRTSALLAGLERAALPLARLGVNPARVSLAVSLTLRFIPV  149

DR_A0270     301  AGGLHPADAVALVTSQPAEYWGLHDLGVIAPGYHADFVLLRDLQHFGVLETFVGGEEARPGGETPPLPGGGVDLGPGWDGATFDPPAHWPTLQMFPDQIV  400
                  ||||||||||||||||||||||||||||||||||||||||||||||||||||||||||||||||||||||||||||||||||||||||||||||||||||
KDR_A0270    301  AGGLHPADAVALVTSQPAEYWGLHDLGVIAPGYHADFVLLRDLQHFGVLETFVGGEEARPGGETPPLPGGGVDLGPGWDGATFDPPAHWPTLQMFPDQIV  400
                                     |||||||||||||||||||||||||||||||||||||||||||||||||||||||||||||||||||||||||||||||||
DR_RS17005   150  VAQTVQDVREAQRARGIEKYWGLHDLGVIAPGYHADFVLLRDLQHFGVLETFVGGEEARPGGETPPLPGGGVDLGPGWDGATFDPPAHWPTLQMFPDQIV  249

DR_A0270     401  TGRAAPGSGDARLVVADRYGRGEWSSCWTLGSGLRGGTLGISILHDAHQVALLGGSDADLRAAGRALERLGGGIVLVVDGEVREQLPLPYAGLMTDHAPA  500
                  ||||||||||||||||||||||||||||||||||||||||||||||||||||||||||||||||||||||||||||||||||||||||||||||  ||
KDR_A0270    401  TGRAAPGSGDARLVVADRYGRGEWSSCWTLGSGLRGGTLGISILHDAHQVALLGGSDADLRAAGRALERLGGGIVLVVDGEVREQLPLPYAGLMTDQPPA  500
                  ||||||||||||||||||||||||||||||||||||||||||||||||||||||||||||||||||||||||||||||||||||||||||||||||||||
DR_RS17005   250  TGRAAPGSGDARLVVADRYGRGEWSSCWTLGSGLRGGTLGISILHDAHQVALLGGSDADLRAAGRALERLGGGIVLVVDGEVREQLPLPYAGLMTDQPPA  349

DR_A0270     501  EAAAALGRVTAAARLLGCMPAGRL                                                                              524
                  ||||||||||||||||||
KDR_A0270    501  EAAAALGRVTAAARLLGCTLPYPVTTLSFLGLSVIPALKLTPRGLLDVTAWRLLDREG                                            558
                  ||||||||||||||||||||||||||||||||||||||||||||||||||||||||||
DR_RS17005   350  EAAAALGRVTAAARLLGCTLPYPVTTLSFLGLSVIPALKLTPRGLLDVTAWRLLDREG                                            407
```

Figure S8. Comparison of amino acid sequences of KDR_A0270 to DR_A0270 and DR_RS17005. The C-terminal segment of KDR_A0270, spanning amino acids 497 to 558, shows alignment with the corresponding region of DR_RS17005, amino acids 346 to 407, previously known as DR_A0268.

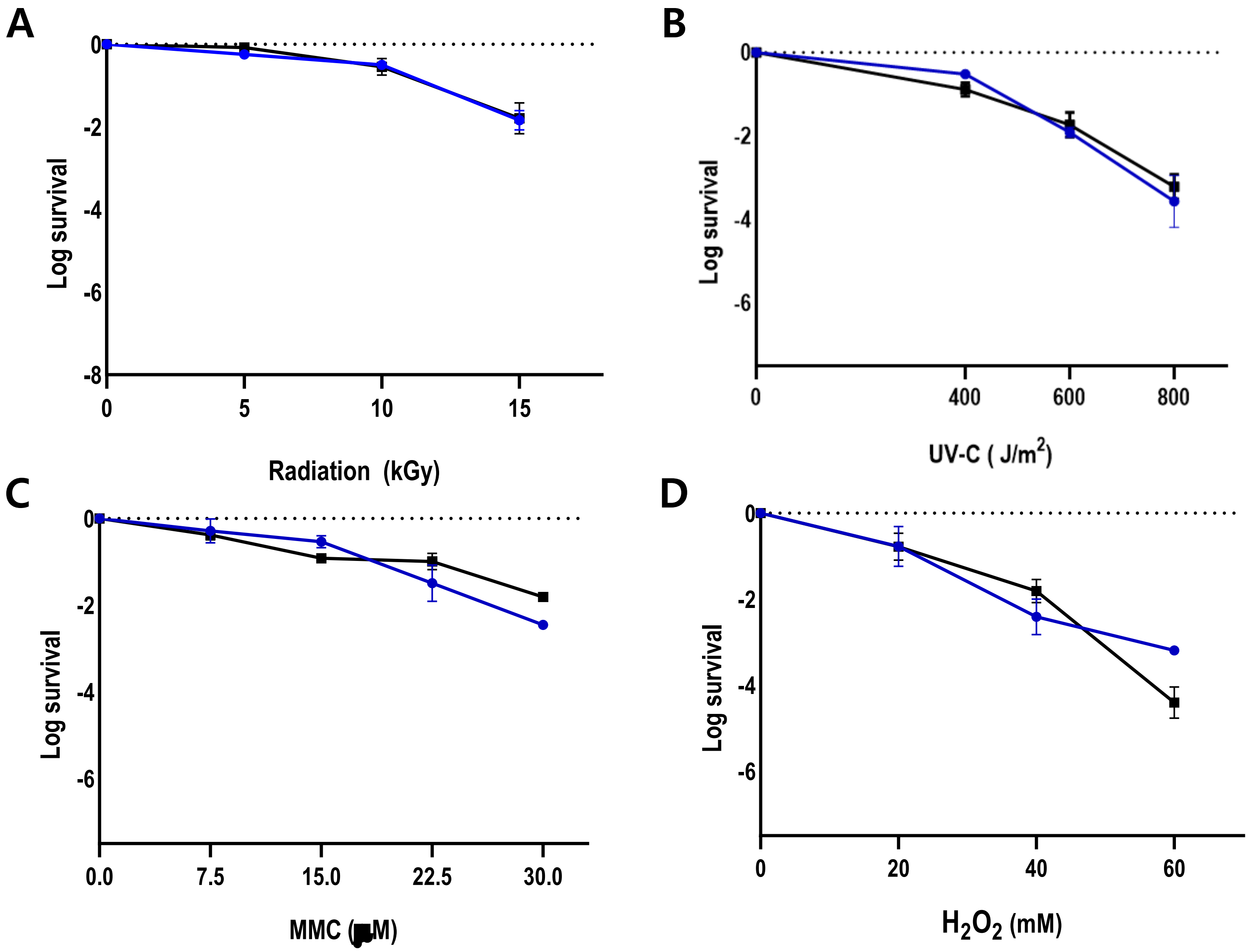

Figure S9. Survival curves for *Deinococcus radiodurans* strains ATCC 13939K (blue lines) and BAA-816 (black lines) following exposure to various stressors. Cells were cultured to logarithmic phase before exposure to specified doses of γ-radiation (A) and ultraviolet-C (B), and concentrations of mitomycin C (C) and hydrogen peroxide (H_2O_2) (D). Following treatment, cells were plated on TGY agar and serial dilutions were performed to determine survival rates. Data points represent mean values from duplicate experiments.

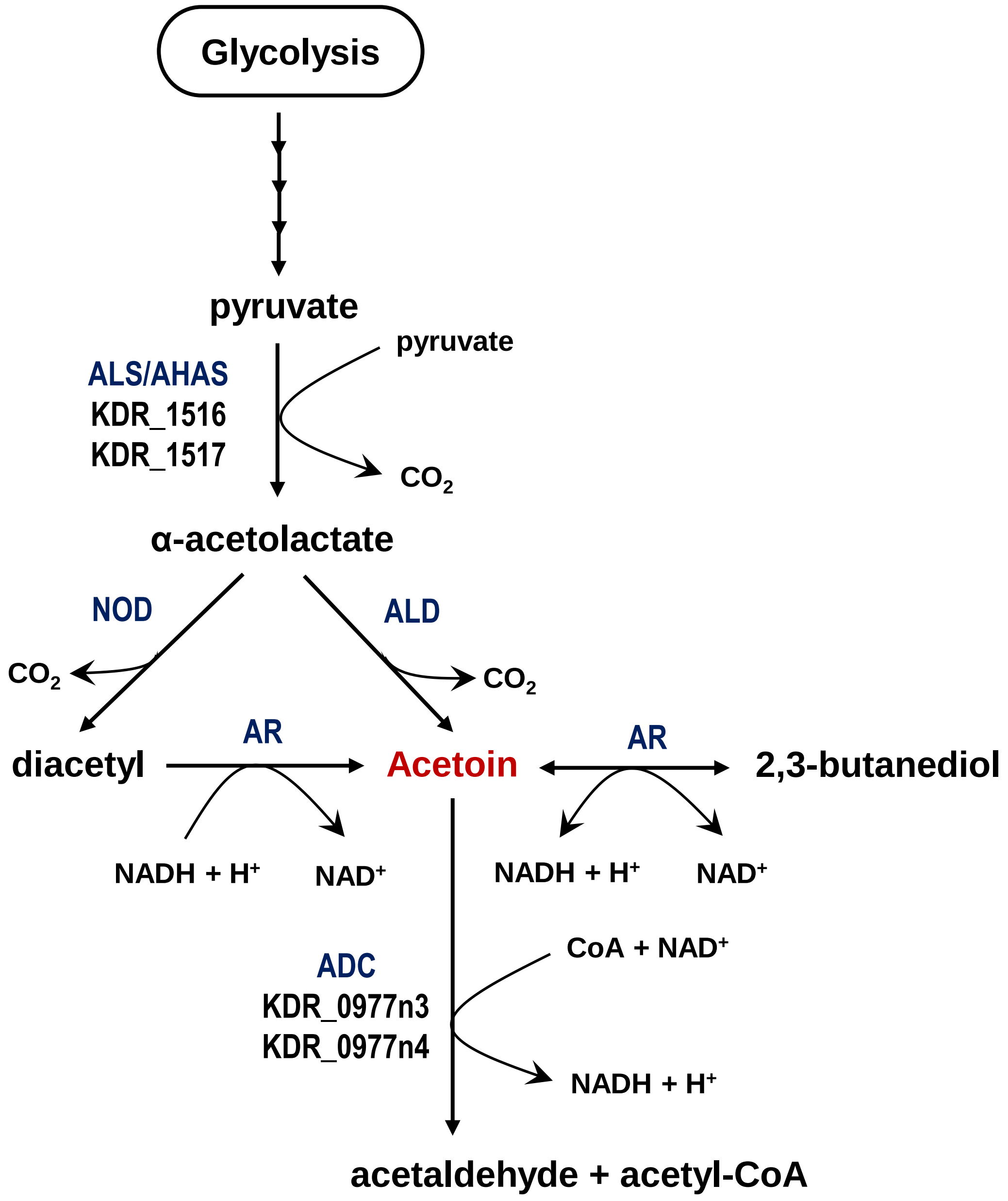

Figure S10. A schematic overview of bacterial acetoin metabolic pathways. Two pyruvate molecules are transformed into acetoin by α-acetolactate synthase (ALS) or α-acetohydroxy acid synthase (AHAS), followed by α-acetolactate decarboxylase (ALD). Alternatively, α-acetolactate may oxidatively decarboxylate into diacetyl, which then becomes acetoin through the action of acetoin reductase (AR). AR also facilitates the reduction of acetoin to 2,3-butanediol. The acetoin dehydrogenase complex (ADC) can break down acetoin into acetaldehyde and acetyl-CoA. The putative AHAS and ADC proteins in *D. radiodurans* are shown.
